# The Microbiome–Neurodegeneration Interface: Mechanisms, Evidence, and Future Directions

**DOI:** 10.3390/cells15020135

**Published:** 2026-01-12

**Authors:** Lilia Böckels, Daniel Alexa, Dorin Cristian Antal, Cristina Gațcan, Cosmin Alecu, Kristina Kacani, Raul Andrei Crețu, Emanuel Andrei Piseru, Robert Valentin Bîlcu, Dan Iulian Cuciureanu

**Affiliations:** 1 University of Medicine and Pharmacy “Grigore T. Popa”, 16 Universității Street, 700115 Iași, Romania; alexadaniel2004@yahoo.com (D.A.); d.antal@alfred.org.au (D.C.A.); gatcancristina@yahoo.com (C.G.); c.raulandy@yahoo.ro (R.A.C.); cuciureanudan@yahoo.com (D.I.C.); 2 Department of Neurology, Rehabilitation Hospital, 700661 Iași, Romania; 3 Department of Neurology, Alfred Health, Melbourne, VIC 3004, Australia; 4 Department of Neurology, Centre Hospitalier Universitaire de Nice, 060000 Nice, Franceconstantin-kacani.k@chu-nice.fr (K.K.); 5 Department of Biomedical Sciences, Faculty of Medical Bioengineering, University of Medicine and Pharmacy “Grigore T. Popa”, 700588 Iași, Romania; 6 Doctoral School, University of Medicine and Pharmacy “Grigore T. Popa”, 700454 Iași, Romania; robert-valentin.bilcu@d.umfiasi.ro; 7 Neurology Department I, “Prof. Dr. N. Oblu” Emergency Clinical Hospital, 2 Ateneului Street, 700309 Iași, Romania

**Keywords:** gut microbiota, neurodegenerative diseases, dysbiosis, microbiome, central nervous system, enteric nervous system, microbiota-gut-brain axis, blood-brain barrier

## Abstract

The gut microbiota has emerged as a central regulator of the gut–brain axis, profoundly influencing neural, immune, and metabolic homeostasis. Increasing evidence indicates that disturbances in microbial composition and function contribute to the onset and progression of neurodegenerative diseases (NDs) through mechanisms involving neuroinflammation, oxidative stress, and impaired neurotransmission. Gut dysbiosis is characterized by a loss of microbial diversity, a reduction in beneficial commensals, and an enrichment of pro-inflammatory taxa. These shifts alter intestinal permeability and systemic immune tone, allowing microbial metabolites and immune mediators to affect central nervous system (CNS) integrity. Metabolites such as short-chain fatty acids (SCFAs), tryptophan derivatives, lipopolysaccharides (LPS), and trimethylamine N-oxide (TMAO) modulate blood–brain barrier (BBB) function, microglial activation, and neurotransmitter synthesis, linking intestinal imbalance to neuronal dysfunction and cognitive decline. Disruption of this gut–brain communication network promotes chronic inflammation and metabolic dysregulation, key features of neurodegenerative pathology. SCFA-producing and tryptophan-metabolizing bacteria appear to exert neuroprotective effects by modulating immune responses, epigenetic regulation, and neuronal resilience. The aim of this work was to comprehensively explore the current evidence on the bidirectional communication between the gut microbiota and the CNS, with a focus on identifying the principal molecular, immune, and metabolic mechanisms supported by the strongest and most consistent data. By integrating findings from recent human studies, this review sought to clarify how microbial composition and function influence neurochemical balance, immune activation, and BBB integrity, ultimately contributing to the onset and progression of neurodegenerative processes. Collectively, these findings position the gut microbiota as a dynamic interface between the enteric and CNS, capable of influencing neurodegenerative processes through immune and metabolic signaling.

## 1. Introduction

Neurodegenerative diseases (NDs) are defined by the progressive dysfunction and loss of neurons in the brain and spinal cord. These conditions represent a growing public health challenge, particularly in aging societies, due to their high rates of disability, morbidity, and mortality. Neurodegeneration is primarily driven by the gradual deterioration of neuronal function, ultimately leading to cerebral atrophy and widespread systemic impairment [1,2].

As the global population ages, the prevalence of NDs is increasing and with advancing age there are some major shifts in gut microbiota, including reduced diversity, impaired intestinal barrier function, and chronic low-grade inflammation, all of which contribute to age-related disorders [3].

The human microbiome, particularly within the gut and oral cavity, far exceeds the human genome in both cell number and genetic content. Microbial cells may account for up to 90% of all human-associated cells, while microbial genes represent nearly 99% of unique genes, leading to the concept of the microbiome as a “second genome” [4,5]. Over 99% of the genetic material linked to the human body is microbial, forming a vast reservoir essential for immunity, homeostasis, and the communication between the central nervous system (CNS) and the enteric nervous system (ENS) [6,7].

The brain–gut axis, a bidirectional communication system between the CNS and gut microbiota, has become a key research focus. Dysbiosis has been associated with neuropsychiatric disorders and may influence processes linked to neurodegenerative disease onset and progression through bioactive metabolites, such as neuropeptides, and immune modulators, that influence neural signaling, behavior, and neuroinflammation [8,9,10,11]. However, human evidence remains largely correlative, with causal relationships primarily inferred from preclinical models. These findings have led to the concept of the microbiome-gut–brain axis (MGBA), a bidirectional network linking the gut microbiota and the CNS via immune, neural, and endocrine pathways. This framework helps explain how gut microbes can influence neurological health and disease [10,11,12,13]. Gut microbiota composition is dynamic, shaped by genetics, environment, lifestyle, diet, and medications [11,12]. Unlike the relatively stable human genome, the microbiome remains adaptable and modifiable, making it an attractive target for therapeutic strategies [14,15].

## 2. Gut Microbiome Overview

Gut microbiota is involved in nervous system development and function throughout life, in older adults, dysbiosis and reduced microbial diversity have been associated with neurodegenerative and neuroinflammatory conditions, including Alzheimer’s disease (AD), Parkinson’s disease (PD), multiple sclerosis (MS) and various motor neuron diseases such as amyotrophic lateral sclerosis (ALS), progressive bulbar palsy, and spinal muscular atrophy [16,17,18,19].

Acquired from maternal and environmental sources, the gut microbiota establishes primarily in the gastrointestinal tract (GIT) and contributes to immune maturation, gastrointestinal (GI) development, CNS regulation, and key metabolic pathways [13,20].

The gut microbiome, the most studied human microbiome, contains nearly 3000 bacterial species grouped into 11 phyla: *Bacillota*, *Bacteroidota*, *Actinobacteria*, and *Proteobacteria* represent over 90% [21,22]. They often form biofilms and coordinate activity through quorum sensing (QS), a communication system using autoinducers that promotes microbial cooperation, biofilm stability, and survival in the host environment [1].

**Figure 1:**
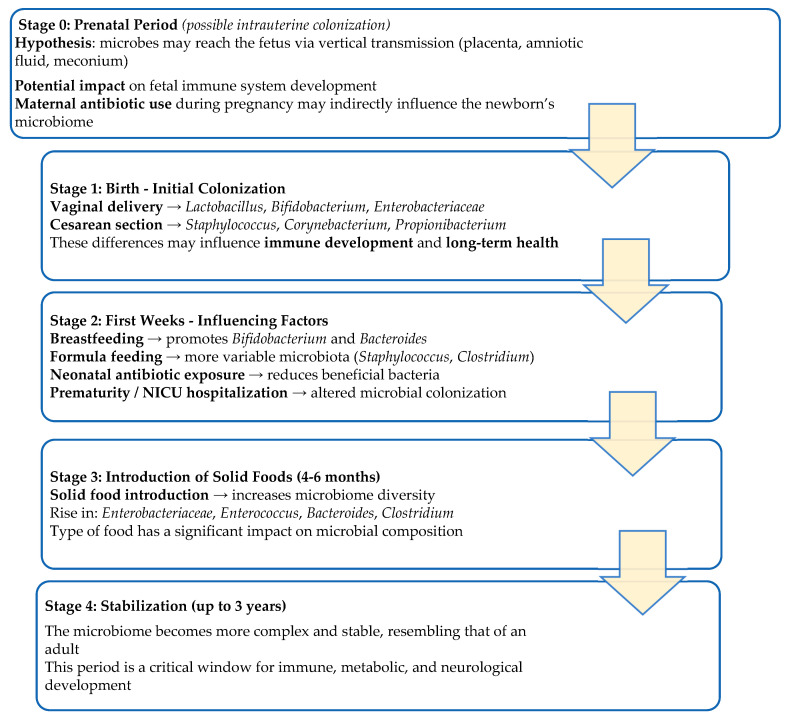
Stages of infant microbiota colonization (from intrauterine life to 3 years): NICU, Neonatal intensive care unit [22].

The human microbiota begins developing early in life (Figure 1). Contrary to the belief that the intrauterine environment is sterile, evidence suggests that the fetus encounters bacteria in utero, potentially initiating microbial colonization and immune programming before birth [23].

Within the first six weeks of life, microbial communities diversify rapidly and become site-specific, with distinct profiles identified in the nostrils, oral cavity, skin, stool, and vaginal regions. This indicates that niche specialization of the microbiota occurs very early. The first two years of life are critical for microbiota establishment, as immune and metabolic systems continue to exhibit significant flexibility. Mode of delivery, antibiotic exposure, and feeding type strongly shape the neonatal microbiota. Vaginal delivery promotes colonization by *Lactobacillus*, *Bifidobacterium*, and *Enterobacteriaceae*, reflecting maternal microbiota, while cesarean section favors *Staphylococcus*, *Corynebacterium*, and *Propionibacterium*, often leading to reduced *Bifidobacterium* and *Bacteroides* levels that may persist long-term [23,24]. Premature birth and low birth weight can significantly alter gut colonization, often due to antibiotic use, neonatal intensive care unit (NICU) care, and cesarean delivery. Feeding mode is another critical factor: breastfeeding promotes the dominance of *Bifidobacterium* spp., supported by human milk oligosaccharides (HMOs) that act as prebiotics, while formula feeding is linked to a more diverse but less stable microbiota, with higher levels of *Staphylococcus*, anaerobic *Streptococcus*, and *Clostridium* [24,25,26,27]. Breast milk also provides live bacteria from multiple genera, further supporting microbiota maturation. Antibiotics given during or after birth can disrupt this balance, reducing beneficial species and favoring opportunists [22].

As infants transition from a milk based diet to solid foods (around 4–6 months), the gut microbiota shifts from *Bifidobacterium* dominance to greater diversity with *Bacteroides* spp. and other carbohydrate degrading bacteria. This diversification, generally completed by age three, marks a key milestone in establishing a stable, adult like microbiota [27,28]. Overall, the human body hosts diverse microbial communities, with the gut microbiota being the most complex and metabolically active, essential for digestion, immunity, and neurodevelopment [24].

While delivery mode and feeding method are well recognized modulators of the early life microbiota, maternal diet, health, and gestational age also play important roles. Diets rich in vegetables and fiber have been associated with higher *Bifidobacterium* abundance, whereas high fat or low plant diets correlate with reduced *Bacteroides*. Such effects may reflect changes in maternal microbiota, altered vertical transmission, or a higher likelihood of cesarean delivery. Specific nutrients, such as red meat derived Neu5Gc, may also influence human milk HMO composition, indirectly shaping infant microbial communities [10,25].

Maternal health conditions, including gestational diabetes, obesity, stress, asthma, and autoimmune or infectious diseases, can further affect microbial colonization. Prenatal stress has been linked to elevated *Proteobacteria* in infants, a marker of dysbiosis and inflammation, while maternal asthma or chronic inflammation is associated with reduced *Lactobacillus* and *Bifidobacterium*, suggesting impaired microbial seeding or altered immune priming [26].

Prematurity adds another layer of disruption. Infants born before 35 weeks are frequently exposed to antibiotics and NICU environments, often missing critical exposure to maternal vaginal and fecal microbiota. Their gut microbiota show delayed colonization of beneficial taxa, reduced diversity, and greater prevalence of opportunists such as *Staphylococcus* and *Enterobacter*, alongside reduced levels of short chain fatty acids (SCFAs), indicating metabolic immaturity. These alterations may persist and are increasingly linked to adverse neurodevelopmental and immune outcomes. The first three years of life represent a critical window for microbiota establishment, with long-term implications for metabolic, immune, and neurological health [22,29,30].

The gut microbiota plays an important role in neuroimmune regulation and has been associated with neurodegenerative diseases, offering potential for microbiome-based preventive and therapeutic strategies [31,32,33]. Evidence from human studies remains largely correlative, with causal relationships primarily inferred from preclinical models. Dysbiosis has been linked to altered maturation and function of CNS immune cells such as microglia, affecting neuroprotection and inflammation, as observed in AD and MS [34,35,36]. Compromised gut barrier integrity can increase permeability, allowing microbial products like lipopolysaccharides (LPS) to enter circulation and weaken the blood–brain barrier (BBB). This dual barrier disruption may connect intestinal imbalance with neurodegeneration by amplifying chronic inflammation [28,37,38].

Microbial metabolites including SCFAs, tryptophan derivatives, and secondary bile acids also influence neurotransmission, microglial activity, and neuronal resilience, linking microbial ecology to core neurodegenerative processes [39,40]. Individual variability in microbiota composition, shaped by genetics and environment, may underlie differing susceptibility to neurological disease. Personalized microbiome profiling could help identify at-risk individuals and guide targeted interventions [41,42]. Notably, human evidence is strongest for immune and barrier-related mechanisms, while links to neurodegenerative outcomes remain less consistent and highly heterogeneous across studies.

## 3. The Gut Microbiota: Composition and Key Functions

Recent work on the brain–gut–bone axis has added further complexity to the understanding of gut–brain interactions, offering new but still preliminary perspectives for the treatment of NDs. Evidence suggests that microbe-associated molecular patterns (MAMPs), including LPS, flagellin, and bacterial DNA, can activate innate immune cells and trigger cytokine release (e.g., TNFα, IL-6, IL-1β). These cytokines may subsequently cross the BBB, potentially altering neuronal and microglial activity. Most of these findings are derived from animal models, and direct evidence in humans remains limited, making the translational relevance of such mechanisms still uncertain [39].

The MGBA is a bidirectional network integrating neural, endocrine, immune, and metabolic pathways [34]. The vagus nerve (VN) represents a major communication route, as shown by findings that vagotomy abolishes the central effects of *Lactobacillus rhamnosus* in rodents and reduces neurological risk in humans when performed early in life [40]. Nevertheless, the translational relevance of these observations remains uncertain, as the human data are largely correlational and may be confounded by clinical and environmental factors. Although several microbial species produce neurotransmitters such as γ-aminobutyric acid (GABA), serotonin (5-HT), dopamine, and acetylcholine, current evidence suggests that these molecules act mainly through modulation of the ENS rather than by directly crossing the BBB [41]. Microbial metabolites such as SCFAs may also influence central processes through epigenetic mechanisms, while gut-derived cytokines (e.g., IL-1, IL-6) can access brain regions with incomplete barrier function, thereby activating the hypothalamic–pituitary–adrenal (HPA) axis and potentially linking intestinal inflammation to stress regulation [42,43,44,45]. Most of these mechanistic insights originate from animal studies, and their significance in humans requires further validation through well controlled clinical investigations.

Microbial dysregulation at both ends of the lifespan appears relevant to brain health. In neonates, a low diversity microbiota dominated by *Proteobacteria* and *Actinobacteria* gradually shifts toward communities enriched in *Bacillota* and *Bacteroidota.* In contrast, preterm infants often display delayed and pathogen prone colonization patterns that have been associated with increased neurodevelopmental risk [27,28,29,30,31,32,33]. In older adults, particularly those living in institutional care, reduced microbial diversity has been correlated with poor diet, cognitive decline, and reduced physical activity. Together, these observations suggest that alterations in microbiota composition across life stages may be associated with changes in brain function. However, most findings are correlative, and causality remains to be established [43]. Importantly, human intervention studies remain heterogeneous, with variable outcomes depending on strain, dose, population, and study design, limiting firm conclusions regarding clinical efficacy [38,42,43,44,45,46,47,48,49]. Nevertheless, these effects are often modest, strain specific, and limited by small sample sizes and short intervention periods. While microbiome targeted strategies hold therapeutic promise, their efficacy and long term safety require more rigorous evaluation in large-scale, placebo-controlled trials.

### 3.1. The Vagus Nerve (VN) and Autonomic Nervous System (ANS)

The gut is innervated by several neuronal systems beyond the VN, including the ENS, dorsal root ganglia (DRG), sympathetic ganglia (SG), and sacral spinal neurons. The ENS, derived from the neural crest, is the largest component of the peripheral nervous system, organized into myenteric and submucosal plexuses that span the gut [48]. Despite constant mechanical, chemical, and microbial stress, the ENS remains structurally stable through continuous regeneration by nestin and enteric neural precursor cells. From an evolutionary standpoint, it is considered the earliest form of a nervous system, predating the CNS, and it continues to interact with the brain via the GBA [49].

The VN, composed predominantly of afferent fibers, serves as the major bidirectional conduit between the gut and the brain, regulating GI functions while transmitting signals that can influence mood, satiety, and immune tone [50,51]. Disrupted vagal signaling has been implicated in mood and metabolic disorders, whereas interventions targeting this pathway have shown variable outcomes in humans [52]. Epidemiological cohort studies suggest that truncal vagotomy may confer partial protection against PD, with one Danish registry reporting a hazard ratio (HR) of 0.53 (95% CI 0.28–0.99) after more than 20 years of follow-up, whereas selective or overall vagotomy showed no significant association (HR 0.85, 0.63–1.14) [40]. Tysnes et al. (2015) re-examined the original Danish data and concluded that the apparent protective association between vagotomy and PD was not statistically robust after controlling for confounding and extended follow-up, emphasizing the methodological limitations of previous analyses [53]. A Swedish matched cohort found a non-significant trend toward reduced PD risk after truncal vagotomy (HR 0.59, 0.37–0.93 for ≥ 5 years) [54], while a Taiwanese nationwide study reported no association with overall dementia incidence (HR 1.09, 0.87–1.36) [55]. More recently, Bunyoz et al. (2022) analyzed 64 000 individuals in a Danish nationwide register and found no protective effect for psychiatric disorders overall, but rather a slightly increased risk after truncal vagotomy (HR 1.22; 95% CI 1.06–1.41), with no effect for highly selective procedures (HR 0.98; 0.84–1.15) [56]. These population data indicate potential long term neuroprotective effects limited to specific surgical subtypes and time frames, though confounding and lack of mechanistic resolution preclude causal inference.

Beyond surgical disruption, vagus nerve stimulation (VNS) has emerged as a therapeutic strategy with measurable immune and neurobehavioral effects in humans. In rheumatoid arthritis, implanted VNS devices significantly reduced circulating TNF, IL-1β, and IL-6, correlating with clinical improvement in DAS28-CRP scores (r = 0.384, *p* < 0.0001 for TNF; r = 0.707, *p* = 0.002 for IL-6) [57]. Open-label pilot trials in Crohn’s disease demonstrated clinical remission in 5 of 9 patients (55.6%) and endoscopic remission in 6 of 9 (66.7%) after 12 months of implanted VNS, accompanied by decreases in CRP and fecal calprotectin [58]. Collectively, human evidence for VN involvement is strongest in inflammatory and mood-related outcomes, whereas associations with neurodegenerative diseases remain inconsistent and context dependent, highlighting the need for mechanistically informed trial designs. Additional neural pathways also contribute to gut–brain communication. DRG neurons transmit nociceptive and immune signals, while sympathetic and sacral nerves provide complementary spinal inputs [51,59]. The ANS, comprising sympathetic and parasympathetic branches, operates in concert with the HPA axis to regulate motility, secretion, biliary flow, and mucosal immunity [56,57].

Microbial molecules including LPS, bacterial amyloids, and metabolites can activate toll like receptor (TLR4) pathways, which have been associated with increased intestinal and BBB permeability and neuroinflammatory responses, though direct evidence in humans remains limited. Probiotics may counteract these effects by strengthening tight junction (TJ) integrity and maintaining barrier function, though direct human evidence remains scarce [58,59,60]. Diet induced dysbiosis further influences gut–brain signaling, particularly through vagal afferent pathways. In rodents, high-fat or low fiber diets remodel the intestinal microbiome, altering the production of SCFAs and bile acids, and generating a low-grade inflammatory environment that compromises vagal neuronal integrity.

These metabolic alterations exert significant effects on vagal afferent signaling, both by modulating enteroendocrine cell activity and by triggering local immune responses within the intestinal mucosa. The resulting low grade inflammatory milieu along the gut-vagus axis may compromise vagal afferent integrity and has been linked to neuroimmune activation. In rodent models, even short-term exposure to a high fat diet induces inflammatory and structural alterations in the colon, nodose ganglion, and hypothalamus, indicating early vagal involvement in diet driven inflammation [61,62,63,64,65].

Dysbiosis associated with high fat feeding alters the morphology and function of vagal afferent terminals, leading to reduced synaptic connectivity and impaired signal transduction between the gut and brainstem. This remodeling represents an adaptive but maladaptive response to chronic metabolic stress, potentially underlying impaired satiety signaling and altered autonomic tone characteristic of metabolic disorders. Mechanistically, such diets reduce vagal mechanosensitivity and blunt responsiveness to gut derived hormones such as cholecystokinin and glucagon-like peptide-1, further weakening vagal mediated regulation of feeding and energy balance [66].

At the cellular level, inflammation and microbial metabolites act synergistically to alter the electrophysiological properties of nodose ganglion neurons. Exposure to LPS and other bacterial components modifies ion channel expression, reducing neuronal excitability and slowing signal conduction along vagal afferents. These effects are accompanied by microglial and astrocytic activation within the nucleus tractus solitarius (NTS), the principal vagal integration center in the brainstem [67,68]. Although these mechanisms have been demonstrated primarily in animal models, emerging human data support partial translational relevance, adherence to a mediterranean style diet rich in fiber and fermentable substrates has been associated with increased plasma SCFAs, enhanced heart rate variability (an indirect marker of vagal tone), and reduced systemic inflammatory markers (CRP, IL-6) [69]. Conversely, high fat Western diets correlate with diminished vagal activity, impaired gut barrier function, and higher circulating LPS levels in human cohorts [70]. These findings suggest that diet-induced shifts in microbial metabolites and inflammation may modulate vagal signaling in humans, although causal pathways remain to be established through mechanistic and interventional studies.

### 3.2. ENS and Microbiota-Neuron Crosstalk

The ENS, often referred to as the “second brain,” is organized into submucosal and myenteric plexuses that regulate motility, secretion, and fluid exchange [62]. It integrates input from vagal and spinal fibers, enabling CNS modulation of gut activity [71]. Microbial metabolites such as SCFAs, 5-HT, and aryl hydrocarbon receptor (AhR) ligands influence ENS function, linking microbial activity to motility and neuroimmune regulation (Table 1) [72]. Enteric microglia, derived from circulating macrophages, act as local immune sentinels and, when chronically activated, contribute to inflammation and gut–brain immune crosstalk [73].

The ANS complements ENS-CNS signaling through sympathetic and parasympathetic branches, regulating motility, barrier integrity, secretion, and immunity [90]. Microbial-derived neurotransmitters, including GABA, 5-HT, and catecholamines, interact with gut neurons and influence brain function via vagal afferents [91]. The VN, projecting mainly to the NTS, connects to brain regions involved in emotion, stress, and cognition. Vagal stimulation promotes hippocampal neurogenesis, brain derived neurotrophic factor (BDNF) expression, and cognitive performance, whereas vagotomy disrupts these processes [66,67,68].

The ANS complements ENS-CNS signaling through its sympathetic and parasympathetic branches, regulating motility, barrier integrity, secretion, and mucosal immunity. Microbial-derived neurotransmitters, including GABA, 5-HT, and catecholamines, interact with gut neurons and influence brain function via vagal afferents [75,76,77]. Microbiota are also essential for ENS maturation: studies in germ-free and TLR-deficient mice show impaired motility, neurochemistry, and 5-HT production, while ENS dysfunction itself can shift the gut microbiota toward a pro-inflammatory profile, illustrating a bidirectional feedback loop [72]. The HPA axis further integrates stress responses, with glucocorticoids shaping neurodevelopment, cognition, and immunity. Dysregulation, reflected in elevated cortisol, is linked to cognitive decline and AD risk, whereas probiotic modulation can reduce stress hormone levels and related effects [70,71,72].

Microbiome-targeted interventions have provided mechanistic evidence, largely from preclinical studies, that modifying the gut microbial ecosystem may influence enteric and CNS activity. Dietary strategies rich in fermentable fibers increase SCFA production, which exerts anti-inflammatory and neurotrophic effects along the GBA. These metabolites have been shown to modulate vagal afferent excitability, intestinal barrier function, and inflammatory signaling, processes implicated in gut–brain communication [92].

Probiotic and prebiotic supplementation also modulates gut–brain signaling at multiple levels. Strains of *Lactobacillus* and *Bifidobacterium* have been reported, primarily in preclinical models, to modulate enteric neuron activity, microglial activation, and the expression of neurotrophic and neurotransmitter molecules such as BDNF and 5-HT. A recent review highlights how probiotic interventions modulate inflammatory signaling cascades, including the NF-κB and MAPK pathways, and restore synaptic plasticity across gut–brain neural circuits [33,87,93]. Fecal microbiota transplantation (FMT) offers additional mechanistic insights, supporting associative and context-dependent links between the microbiome and neuronal function. Transplantation from healthy donors has been shown to ameliorate dysbiosis, attenuate neuroinflammatory markers, and restore gut–brain neurotransmission, particularly in animal models, with limited and heterogeneous evidence in clinical studies. Complementary analyses indicate that FMT can rebalance immune signaling, normalize vagal tone, and improve behavioral and cognitive outcomes in disorders linked to microbiome dysregulation [94,95,96,97,98,99,100]. Overall, human evidence is currently strongest for immune and barrier-related effects, whereas direct modulation of CNS function remains largely supported by preclinical data, underscoring the need for mechanistically informed human trials.

## 4. Pathways of Gut–Brain Communication

Within the gut, regulatory loops link microbiota, enterochromaffin cells, and enteric neurons through biogenic amine signaling to maintain homeostasis [88,89]. Most of the mechanistic understanding of this interplay derives from preclinical models, which have shown that disruption of these signaling pathways is associated with impaired enteric neuronal activity and altered tight-junction integrity. Such alterations have been linked to increased intestinal permeability. Such alterations increase intestinal permeability, allowing translocation of proinflammatory cytokines such as IFN-γ, TNF-α, IL-1β, and IL-17, while reducing anti-inflammatory mediators like IL-10 [92,93,94,95,96]. In animal and in vitro studies, barrier dysfunction has been linked to dysbiosis, impaired regulatory T cell (Treg) differentiation, and a proinflammatory Th1/Th17 shift [101,102,103,104,105]. Reduced SCFA production particularly butyrate further weakens mucosal immunity by lowering secretory IgA and antimicrobial peptide levels, as demonstrated in murine models of diabetes and malnutrition [106,107,108,109,110].

Loss of epithelial integrity may facilitate systemic translocation of microbial components such as LPS, which can trigger low-grade inflammation and immune activation extending to the CNS. Evidence from preclinical studies shows that enteric microglia within Auerbach’s and Meissner’s plexuses respond to inflammatory signals by adopting macrophage-like phenotypes, while circulating monocytes may infiltrate the ENS, creating a bidirectional immune circuit [73,111,112,113]. Zebrafish models carrying sox10 mutations have provided additional evidence that structural ENS disruption can provoke microbiota-driven inflammation, emphasizing how developmental genetic defects alter the gut-immune interface [114]. Chronic microglial activation observed in rodent studies increases peripheral macrophage trafficking toward the NTS, a phenomenon thought to affect autonomic and cognitive function [115]. While these findings offer valuable mechanistic insights, they remain largely preclinical, and their translational relevance to human neuroimmunology is still uncertain.

Human observational evidence increasingly supports a functional connection between GI and neurological health. Historical clinical observations described the so-called “institutional colon” in psychiatric patients, once attributed mainly to diet or pharmacological effects. More recently, a large retrospective analysis of U.S. Veterans Affairs medical records revealed significantly higher rates of colonic dysmotility among individuals with presenile dementia, AD, PD, ALS, and HD, suggesting that intestinal motility is influenced not only by the ENS but also by vagal and spinal pathways [116]. This large-scale human dataset provides epidemiological support for GBA involvement in neurodegenerative disease, though its retrospective and correlational nature limits causal inference.

NDs are now understood to result from an interplay between intrinsic cellular defects and extrinsic environmental or microbial factors. Intrinsic mechanisms include proteostatic stress, impaired protein degradation via the ubiquitin-proteasome and autophagy lysosomal systems, oxidative stress from excess of reactive oxygen species (ROS), and heritable mutations affecting cellular signaling and resilience [106,107,108,109,110,117,118,119,120].

Extrinsic mechanisms, supported by both animal and human studies, involve immune dysregulation, microbial infections, and chronic dysbiosis, which collectively sustain low-grade inflammation and interfere with neuronal repair pathways [42,48,81]. Age remains the strongest risk factor, as cumulative mitochondrial dysfunction, DNA instability, and systemic inflammation gradually heighten susceptibility. Often, a secondary “hit”such as infection, dietary imbalance, or metabolic stress precipitates disease onset in predisposed individuals, underscoring the multifactorial nature of neurodegeneration [46,121].

## 5. Gut Microbiota Brain Signaling and the Immune System

The immune and CNS are closely interconnected through shared developmental pathways and molecular mediators. Once considered immune-privileged, the brain is now recognized to produce immune-related molecules such as Toll-like receptors (TLRs), cytokines, complement proteins, and MHC molecules, all of which shape neural development and function [122,123]. The discovery of meningeal lymphatic vessels has further revealed direct anatomical connections between the peripheral immune system and the CNS, offering insights into brain autoimmunity. Gut dysbiosis has been associated with alterations in epithelial integrity, including changes in TJ proteins, increased permeability, and translocation of proinflammatory molecules such as LPS into circulation. These processes have been linked to neuroinflammatory responses, involving TNF-α, IL-1β, IFN-γ, and IL-17, alongside reduced IL-10 [111,124].

Dysbiosis has been associated with reduced SCFA production, impaired secretion of IgA and antimicrobial peptides (AMPs), features linked to increased microbial and toxin translocation [101,115]. These intestinal immune signals can reach the NTS, a key brainstem relay sensitive to peripheral inflammation, thereby influencing higher brain regions involved in cognition and behavior [125,126].

The gut immune system maintains both intestinal and CNS homeostasis by distinguishing between harmful and harmless microbial signals through receptors such as TLRs, which detect MAMPs including LPS and peptidoglycan (PGN) [127]. Dysregulation of these signaling pathways has been associated with excessive cytokine release, some of which can cross the BBB and activate microglia, promoting neuroinflammation. Evidence from germ free (GF) and antibiotic treated animal models demonstrates that microbiota depletion impairs immune homeostasis in both the gut and brain, partly through TNFα dependent monocyte migration into the CNS, a process reversible following probiotic recolonization [117,118]. Similarly, Rag1 deficient mice, which lack functional lymphocytes, display cognitive and anxiety like deficits that improve after administration of Lactobacillus rhamnosus and L. helveticus [72,118,119].

The intestinal immune system, comprising macrophages, dendritic cells (DCs), innate lymphoid cells, and adaptive T and B cells, interacts continuously with microbes through antigen recognition and immunomodulatory signaling [128,129,130]. Certain bacterial taxa have been shown in animal studies to drive effector T cell differentiation, facilitating their infiltration into the CNS and the release of proinflammatory cytokines that weaken the BBB and perpetuate neuroinflammation [131]. Microbial metabolites such as LPS and amyloids can also transmit inflammatory cues from the intestine to the CNS, mechanisms particularly relevant in preclinical models of PD [132].

Conversely, human data suggest that CNS injury or neuropsychiatric stress can alter gut microbiota composition and immune profiles, with changes in intestinal permeability and immune activation detectable in patients with AD and PD [123,124]. These observations highlight a bidirectional inflammatory loop in which central or peripheral stress can initiate dysbiosis, sustaining chronic inflammation and neuronal injury, especially in amyloid associated neurodegenerative disorders [133,134,135].

The gut microbiota has been shown, primarily in preclinical models, to modulate key immune pathways involved in neuroimmune communication, including the inflammasome, type I interferon (IFN-I), and NF-κB signaling cascades [19,125]. Preclinical models demonstrate that dysregulated inflammasome activation elevates IL1β, IL6, and IL18, cytokines also elevated in patients with major depressive disorder, suggesting a shared immune signature between peripheral and central inflammation. In MS, human clinical data show that IFNI signaling suppresses inflammasome activation, promotes dendritic cell maturation, and enhances protective T cell responses. Complementary studies reveal that certain lactic acid bacteria can stimulate TLR3 dependent IFNI production in intestinal DCs, linking microbial activity to systemic immune tone [136,137,138,139,140]. In murine colitis models, elevated NF-κB and TNFα expression in both gut and hippocampus correlate with memory deficits, whereas restoration of microbial diversity reverses these changes [37,127].

Microglia, representing roughly 10% of all neural cells, originate from embryonic myeloid progenitors and function in host defense, synaptic remodeling, and neuroimmune regulation [122,123,124]. Their maturation and surveillance capacity depend heavily on gut microbiota composition, as shown in GF mice, where microglial morphology and cytokine profiles are altered and restored following colonization [141]. Translational studies in humans further suggest that altered gut microbial diversity correlates with microglial activation markers in neurodegenerative conditions, although causal mechanisms remain speculative. The microbiome is now recognized as a key modulator of CNS autoimmunity. FMT from MS patients into GF mice increases susceptibility to experimental autoimmune encephalomyelitis (EAE), while supplementation with SCFAs mitigates axonal injury and regulates T cell activity. Other microbial metabolites, including tryptophan derivatives, modulate astrocyte reactivity and CNS inflammation. In human studies, antibiotic induced dysbiosis is associated with reduced hippocampal neurogenesis and cognitive decline, effects partially reversed by probiotic supplementation. These results emphasize that microbial composition and metabolic output are central to neuroimmune equilibrium, but direct causal evidence in humans remains scarce [131,142].

Under physiological conditions, the gut microbiota and host immune system co develop to maintain balanced inflammatory responses. Preclinical research shows that GF rodents exhibit exaggerated stress reactivity and anxiety like behavior, reversible only if colonization occurs early in life, highlighting the developmental role of the microbiome. Microbial components such as LPS and amyloids can activate inflammatory pathways that accelerate neurodegeneration in models of AD [133,143].

The microbiota directly shapes gut-associated lymphoid tissue (GALT), influencing lymphocyte maturation, IgA-producing B cells, and AMP secretion [144,145]. Segmented filamentous bacteria promote Th17 differentiation, which supports mucosal defense but can also contribute to autoimmunity. Microbial metabolites, including SCFAs, tryptophan derivatives, and polyamines, further regulate immune balance by enhancing Treg differentiation and exerting anti-inflammatory effects via NF-κB and histone deacetylase (HDAC) inhibition [135,136,137].

Commensal microorganisms further modulate innate immunity. Macrophages adjust cytokine output (IL10, IL12) in response to *Lactobacillus* strains, promoting tolerance and barrier stability [138,139]. Neutrophils can be dampened by microbial signals, with some *Lactobacillus* species inducing apoptosis through NF-κB suppression. Eosinophils depend on microbiome regulated IL-25 for activation, as shown in *Clostridium difficile* infection, while basophils and mast cells are likely influenced by microbial metabolites and mucosal cytokines [32,140,141,142,143]. NK cells also respond to probiotics, with *Bifidobacterium lactis* enhancing cytolytic activity in elderly subjects [144,145]. The complement system, continuously shaped by the microbiota, requires tight regulation to avoid inappropriate activation against commensals [146,147].

DCs function as key antigen presenting cells at the mucosal interface, discriminating between commensals and pathogens through pattern recognition receptors (PRRs) and tolerance pathways [148,149,150,151,152,153,154,155,156,157]. Microbiota also influences adaptive immunity: SCFAs enhance CD8^+^ T cell cytotoxicity via IFN-γ induction [158], Th17 cells secrete IL-22 to support epithelial integrity but may promote goblet cell loss in coliti [159], while T follicular helper cells (TFHs) regulate B cell activation and antibody class switching. Microbial antigens influence antibody repertoires, IgE levels, and IgA production, all critical for coating commensals and maintaining mucosal balance. Human studies indicate that IgA deficiency correlates with reduced microbiota diversity, underscoring the reciprocal nature of host microbe immune interactions. Early life microbial exposure expands antibody repertoires and enhances immune competence, supporting the concept that microbial diversity during development is essential for long term neuroimmune resilience [152,153,154,155,156,157].

## 6. Neurotransmitters

Communication between the gut microbiota and the brain occurs through interconnected immune, neural, hormonal, and metabolic pathways [158,159,160,161,162]. Microbes may influence CNS-related processes via signaling molecules such as dopamine, 5-HT, GABA, neuropeptides, corticotropin-releasing hormone axis (CRH), SCFAs [156,157].

Beyond neurotransmitters, microbiota derived vitamins and metabolites shape neurological and immune functions, acting in limited cases directly when they cross the BBB, or more commonly indirectly through neuroendocrine, immune, or vagal pathways [163,164,165]. Microbes also modulate enteroendocrine hormone release, epithelial barrier integrity, and microglial activity, processes increasingly associated with to AD, PD, autism spectrum disorder, and MS [166,167,168,169].

The microbiota further regulates brain chemistry by controlling the availability of neurotransmitter precursors [22,23,24,25,161]. Some bacteria encode enzymes required for precursor synthesis, while microbial metabolites can stimulate enteroendocrine cells to release neurotransmitters that act locally on the ENS or relay rapid signals to the brain through the VN [161,162].

Excitatory neurotransmitters such as glutamate, acetylcholine, and dopamine, and inhibitory ones such as GABA, glycine, and 5-HT, are fundamental for movement, mood, memory, and learning [170]. Although neurotransmitters themselves do not cross the BBB, their dietary precursors, including tyrosine and tryptophan, can. Once inside the brain, these amino acids are enzymatically converted into active neurotransmitters, illustrating a potential mechanism by which the gut microbiota, through regulation of precursor synthesis and absorption, may influence brain function and behavior [164,165,171,172,173].

Microbes affect neurotransmission both by producing transmitters directly and by generating precursors and enzymes essential for their synthesis [166]. Their impact on brain signaling occurs mainly through the ENS and rapid vagal communication [174]. Specific strains have been identified as major producers: *Escherichia coli*, *Bacillus cereus* and *Bacillus subtilis* generate norepinephrine and dopamine, *Staphylococcus aureus* converts L-DOPA into dopamine, while *Lactobacillus brevis*, *Lactobacillus plantarum*, *Bifidobacterium dentium* and *Escherichia coli* produce GABA, which has been implicated in hippocampal cognitive functions in preclinical models [168,169]. *Candida albicans* and *Morganella morganii* synthesize histamine, while *Bacillus subtilis*, *Lactobacillus plantarum*, *Escherichia coli*, *Staphylococcus aureus* can produce acetylcholine and converts L-DOPA into dopamine. *Bifidobacterium infantis* elevates plasma tryptophan, the precursor of 5-HT [72,170,171].

Serotonin, which regulates cognition, gut motility, secretion, and circadian rhythm, is primarily produced by enterochromaffin cells in the gut, accounting for about 90% of total body 5-HT. Spore forming *Clostridium* species enhance its synthesis by upregulating tryptophan hydroxylase 1, while microbial metabolites such as SCFAs and bile acids modulate this process. *Staphylococcus aureus* also converts 5 hydroxytryptophan into 5-HT [175,176,177].

GF and antibiotic treated animal models show that the absence of microbiota profoundly alters neurotransmitter levels and receptor expression in the brain, gut, and systemic circulation, underscoring the bidirectional communication between microbes and the host’s neurochemical environment [178]. Glutamate, the most abundant excitatory neurotransmitter, is synthesized in the brain via neuron-astrocyte cooperation and tricarboxylic acid (TCA) cycle intermediates, as it cannot cross the BBB [172,173]. In the gut, specialized enteroendocrine “neuropod cells” also produce glutamate and release it via vesicular glutamate transporter 1 (VGLUT1) transporters to activate vagal pathways, transmitting nutrient signals to the brain within milliseconds [174]. Additionally, microbial fermentation of carbohydrates produces acetate, which can cross the BBB. In animal studies, labeled inulin fermentation resulted in acetate accumulation in the hypothalamus, supporting glutamate glutamine cycling between neurons and glia, suggesting a microbiota-dependent pathway, primarily supported by animal studies [167,174].

GABA, the primary inhibitory neurotransmitter, is synthesized from glutamate by glutamic acid decarboxylase in the CNS [179,180,181,182]. Multiple taxa, including *Bacteroides fragilis*, *Parabacteroides*, *Eubacterium*, and *Bifidobacterium* species, produce GABA. Notably, strain KLE1738 from the *Ruminococcaceae* family depends on *B. fragilis* derived GABA for growth [171]. While GABA itself does not cross the BBB, it may influence the ENS or act through vagal signaling. In addition, microbiota derived acetate can integrate into brain GABA synthesis, particularly in the hypothalamus, paralleling its role in glutamate metabolism. These findings, largely based on preclinical work, demonstrate that microbial GABA production affects neural circuits indirectly through peripheral or vagal routes, though the translation to human physiology remains to be fully elucidated. Acetylcholine serves as a major excitatory neurotransmitter in both the central and peripheral nervous systems and is essential for synaptic communication, attention, and memory, processes that decline in AD. Initially attributed to ergot alkaloids, its microbial source was later identified as *Bacillus acetylcholini* [175,176]. Several gut microbes, including *Lactobacillus plantarum*, *Bacillus subtilis*, *Escherichia coli*, and *Staphylococcus aureus*, can produce acetylcholine, with *B. subtilis* generating higher levels than *E. coli* or *S. aureus*. Other commensals, such as Enterococcus faecium and Clostridium butyricum, have also been shown to release acetylcholine or modulate its biosynthetic pathways. Since acetylcholine cannot cross the BBB, it must be synthesized locally in the brain from dietary choline and acetyl-CoA [177,178,179].

Dopamine, the most abundant catecholamine, is synthesized in the substantia nigra and ventral tegmental area, where it regulates cognition, emotion, motor control, and reward pathways. It is derived from dietary tyrosine, which crosses the BBB and is enzymatically converted to dopamine [183,184,185,186]. In the gut, *Staphylococcus* species produce dopamine by decarboxylating L-DOPA via SadA, while *Bacillus cereus*, *Serratia marcescens*, and *Enterococcus faecalis* can also contribute to catecholamine pool, more than half of total body dopamine, originates outside the brain, where it primarily regulates peripheral functions such as motility, secretion, and mucosal blood flow [61,160,181]. Human studies show that fecal dopamine levels correlate with gut microbial composition, but causality and CNS effects remain uncertain.

**Figure 2:**
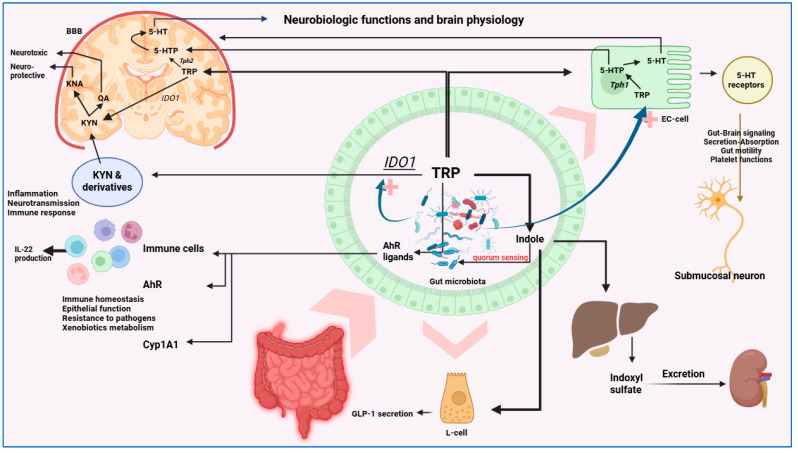
Integrated Trp metabolism under the control of the gut microbiota: Dietary tryptophan (TRP) is metabolized by gut microbiota into aryl hydrocarbon receptor (AhR) ligands that regulate immune balance and intestinal barrier integrity. Microbes also modulate the indoleamine 2,3-dioxygenase (IDO), kynurenine pathway, influencing inflammation, immune signaling, and neurobiological activity. Peripheral 5-HT produced by enterochromaffin cells acts locally to stimulate gut motility and indirectly affects central serotonergic pathways by altering Trp and tryptamine availability. 5-HTP, 5-hydroxytryptophan; IL, interleukin; QA, quinolinic acid; TRP, tryptophan; AhR, aryl hydrocarbon receptor; IDO, indoleamine 2,3-dioxygenase; IDO1, indoleamine 2,3-dioxygenase 1; TPH1, tryptophan hydroxylase 1; TPH2, tryptophan hydroxylase 2; KNA, kynurenic acid; KYN, kynurenine; BBB, blood–brain barrier; CYP1A1, cytochrome P450 1A1; GLP-1, glucagon-like peptide 1.

Serotonin, primarily synthesized in the raphe nuclei, is vital for mood, sleep, and cognition, but approximately 90% of total 5-HT is produced peripherally by enterochromaffin cells using dietary tryptophan (Figure 2) [187,188]. Bacterial metabolism, especially through the kynurenine pathway, influences this process [189]. Spore forming *Clostridia* upregulate TPH1, the rate-limiting enzyme in 5-HT synthesis, while *Staphylococcus aureus* can convert 5-HTP into 5-HT. Microbes also produce trace amines such as tyramine, tryptamine, phenylethylamine, and octopamine. *Providencia* in *Caenorhabditis elegans* generates tyramine, which the host converts into octopamine, altering feeding behavior [190]. In humans, tryptamine from *Clostridium sporogenes* and *Ruminococcus gnavus* stimulates enterochromaffin 5-HT release and enhances gut motility. Gut bacteria such as *Bacillus subtilis*, *Escherichia coli*, and *Serratia marcescens* produce norepinephrine or its precursors. In animal models, exposure to cold stress modifies gut microbiota composition and increases norepinephrine levels in both the intestine and brown adipose tissue, supporting a microbiota dependent mechanism for neuroendocrine adaptation [168].

Although direct evidence in humans is limited, gut derived catecholamines may act locally to influence motility and mucosal perfusion, thereby indirectly affecting systemic stress pathways. Norepinephrine, another major catecholamine, is synthesized in the locus coeruleus from dopamine and plays a central role in arousal, attention, memory, and stress responses [191]. Microbiota composition can affect norepinephrine availability, cold exposure alters gut microbiota and increases norepinephrine in the gut and brown adipose tissue [191,192]. SCFAs have been widely described as beneficial mediators of gut–brain axis signaling; accumulating evidence indicates that their role in neurodegenerative conditions is complex and context dependent rather than uniformly neuroprotective [193]. Meta-analytical and clinical data demonstrate that SCFA alterations reflect an interplay between microbial dysbiosis, gastrointestinal dysfunction, intestinal barrier integrity, and systemic metabolism, rather than a simple deficiency or excess model [194]. SCFAs are generated through microbial fermentation of indigestible dietary fibers, with acetate, propionate, and butyrate accounting for approximately 95% of total SCFA production [195,196]. Importantly, fecal SCFA concentrations represent only a residual fraction, as approximately 90–95% of luminal SCFAs are absorbed by colonocytes or transported into circulation, indicating that stool measurements do not directly reflect systemic exposure [197].

Gastrointestinal dysfunction plays a central role in shaping SCFA profiles. Prolonged intestinal transit and constipation, which affect a large proportion of patients with neurodegenerative disorders, alter microbial composition toward reduced SCFA-producing taxa, particularly butyrate producers, and enhance epithelial uptake of SCFAs [198]. In parallel, compromised intestinal barrier integrity facilitates increased passage of luminal metabolites into the bloodstream, contributing to elevated circulating SCFA levels. While reduced luminal SCFAs have been associated with impaired mucus production, loss of tight junction integrity, and altered immune regulation, increased systemic SCFAs may reflect pathological rather than protective processes. Elevated plasma levels of valeric, isobutyric, and propionic acids have been associated with disease severity and cognitive impairment, indicating divergent effects depending on compartmentalization and concentration [196,199].

Experimental and clinical studies further support this duality. Although SCFAs have been shown to maintain BBB integrity and influence neurogenesis through epigenetic mechanisms, excessive systemic concentrations may exert detrimental effects. Elevated SCFA levels promote neuroinflammation and exacerbate motor dysfunction, dopaminergic neuronal loss, and glial activation in experimental models, particularly when supraphysiological exposure occurs [200]. Mechanistically, SCFAs can activate G-protein-coupled receptors, including GPR41, GPR43, and GPR109A, as well as epigenetic pathways through histone deacetylase inhibition, with downstream effects that vary according to receptor engagement, tissue localization, and inflammatory context [201,202,203].

In particular, recent mechanistic evidence implicates the SCFA/GPR43-NLRP3 inflammasome axis in neuroinflammatory signaling. SCFA exposure has been shown to exacerbate motor and gastrointestinal dysfunction, intensify α-synuclein pathology, and promote dopaminergic neuronal loss through activation of GPR43-dependent NLRP3 inflammasome signaling. Inhibition of either GPR43 or NLRP3 mitigates α-synuclein accumulation, neuronal loss, and inflammatory responses, highlighting a receptor- and pathway-specific mechanism by which SCFAs may contribute to neurodegenerative pathology under specific conditions. SCFAs act locally on gut epithelial cells through FFAR2 and FFAR3 receptors, enter circulation to regulate lipid and glucose metabolism, and cross the BBB via monocarboxylate transporters to influence neuronal metabolism [204].

SCFAs, including acetate, propionate, and butyrate, are produced by bacterial fermentation of dietary fibers such as galacto- and fructo-oligosaccharides. Key producers include *Bacteroides*, *Bifidobacterium*, *Propionibacterium*, *Eubacterium*, *Lactobacillus*, *Clostridium*, *Roseburia*, and *Prevotella* species [205,206]. SCFA profiles vary depending on fiber source and microbial composition. Members of the *Bacillota phylum*, particularly *Roseburia*, *Eubacterium*, and *Lachnospiraceae*, are prolific butyrate producers, whereas *Bifidobacterium* species primarily yield acetate and lactate [188,189,190]. SCFAs act locally on gut epithelial cells through FFAR2 and FFAR3 receptors, enter circulation to regulate lipid and glucose metabolism, and cross the BBB via monocarboxylate transporters to influence neuronal metabolism [207].

Butyrate functions as an HDAC inhibitor, promoting hyperacetylation and transcription of neuroprotective genes, including FOXO transcription factors that regulate antioxidant defense, autophagy, and stress resilience [208]. While FOXO activation supports neuronal survival, dysregulation may favor apoptosis, relevant in PD where mitochondrial dynamics are impaired [209]. Experimental studies show that butyrate protects dopaminergic neurons from α-synuclein-induced DNA damage, reduces amyloid precursor protein (APP) expression, and improves cognition in AD models. Similar neuroprotective effects have been reported for β-hydroxybutyrate in excitotoxicity and protein aggregation models [210]. Similar effects have been reported for β-hydroxybutyrate in excitotoxicity and protein aggregation [211].

**Figure 3:**
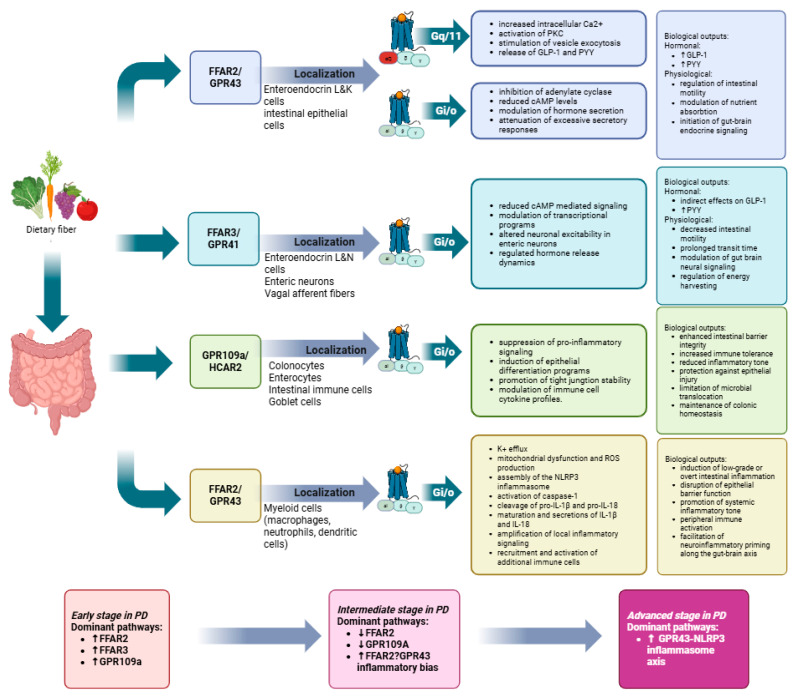
This new schematic illustrates how gut-derived SCFAs exert divergent biological effects depending on their site of action and receptor engagement, distinguishing between protective signaling pathways mediated by FFAR2, FFAR3, and GPR109A at the intestinal and immune interface, and pro-inflammatory signaling mediated by GPR43-NLRP3 inflammasome activation under pathological conditions. By presenting these mechanisms in a unified visual framework, the model reconciles previously conflicting findings regarding SCFA neuroprotective versus neuroinflammatory effects and supports a context-dependent interpretation of SCFA signaling in Parkinson’s disease. This addition directly addresses the reviewer’s request for a mechanistically integrated, evidence-weighted representation of SCFA signaling within the gut–brain axis. SCFAs, short-chain fatty acids; FFAR2, free fatty acid receptor 2; GPR43, G protein-coupled receptor 43; FFAR3, free fatty acid receptor 3; GPR41, G protein-coupled receptor 41; GPR109A, G protein-coupled receptor 109A; HCAR2, hydroxycarboxylic acid receptor 2; NLRP3, NOD-like receptor family pyrin domain-containing 3; PYY, peptide YY; ENS, enteric nervous system; GPCR, G protein-coupled receptor; Gi/o, inhibitory G protein alpha i/o family; Gq/11, G protein alpha q/11 family.

Propionate activates intestinal gluconeogenesis via FFAR3 signaling (Figure 3), relaying satiety and metabolic cues to the dorsal motor nucleus of the vagus (DMV), a site affected early in PD and AD. Butyrate can also stimulate vagal afferents, improving cognition in AD patients [212]. SCFAs further modulate neurotransmitter systems by regulating 5-HT synthesis in enterochromaffin cells, catecholamine production through tyrosine hydroxylase, and dopamine metabolism, processes central to PD [208,209].

These findings indicate that the effects of SCFAs on neurodegeneration are highly context dependent. While balanced production and physiological compartmentalization may confer anti-inflammatory and neuroprotective benefits, dysbiosis, altered host metabolism, increased intestinal permeability, or skewed SCFA ratios can instead promote neuroinflammation and worsen disease outcomes [210,211,212,213,214,215].

## 7. Evidence Linking the Microbiome to Neurodegeneration

The BBB, formed by endothelial cells, TJs (occludin, claudins, JAMs, ZO proteins), pericytes, basement membrane, and astrocytes, regulates nutrient exchange while restricting toxins [216]. TJ alterations contribute to BBB dysfunction in NDs [217,218,219]. Gut-derived metabolites such as LPS, SCFAs, trimethylamines, and vitamins modulate BBB permeability; dysbiosis may impair barrier function via systemic immune activation. In animal models, LPS increased BBB permeability by 60%, while microbiota modulation restored integrity by upregulating claudin-5 and occludin. GF mice also show leakier BBB, corrected after colonization with normal microbiota [213,214].

The intestinal barrier, consisting of mucus (mucin 2) and an epithelial monolayer of specialized cells (goblet, enterocytes, enteroendocrine, microfold, Paneth), relies on tight and adherens junctions [220,221]. Its disruption increases antigen translocation, triggering immune activation and inflammation locally and systemically [109,110]. Barrier dysfunction is linked to AD, PD, obesity, type 1 diabetes, depression, and other systemic conditions [37,217,218]. The intestinal wall, composed of the mucosa, submucosa, muscularis, and serosa, forms a complex barrier that enables selective nutrient absorption while preventing the entry of harmful substances and microorganisms. The term intestinal barrier highlights its protective role, whereas intestinal permeability reflects its functional integrity. When this permeability becomes dysregulated, it can contribute to numerous acquired pathologies [222,223,224,225]. The integrity of the intestinal barrier represents a key determinant of gut and systemic health. This barrier acts as a dynamic interface separating the host from the intestinal lumen and its dense microbial community, while ensuring selective absorption of nutrients and water. Rather than a simple wall, it is a multilayered defense network composed of mucus, epithelial cells, and underlying immune tissue, each contributing uniquely to intestinal homeostasis [226].

The outermost mucus layer, rich in mucin polymers, provides the first line of protection and serves as a habitat for commensal microorganisms, including bacteria, fungi, viruses, and protozoa [227]. The mucus layer is organized into two distinct sublayers: the outer, loose layer that hosts dense communities of commensal microorganisms competing with potential pathogens, and the inner, compact layer that remains largely sterile and physically separates microbes from the epithelial surface. The gut microbiota continuously modulates mucin synthesis and degradation, ensuring the dynamic stability of this barrier. Within this ecological niche, secreted mucins such as MUC2, MUC5AC, and MUC6 form the gel-like scaffold, while transmembrane mucins, MUC1, MUC3, MUC4, MUC13, and MUC17, anchor the mucus to epithelial cells and sustain structural integrity. These components prevent microbial adherence, trap pathogens, and generate antimicrobial molecules that fortify the mucosal defense [228]. When this balanced system is disturbed, a state of dysbiosis emerges, characterized by reduced microbial diversity and dominance of potentially pathogenic species. Such alterations impair epithelial TJs, weaken mucin production, and heighten intestinal permeability, events collectively recognized as leaky gut syndrome [229]. Microbial distribution within the GIT is highly compartmentalized. Upper intestinal regions are enriched in *Pseudomonadota, Streptococcaceae*, and *Veillonellaceae*, whereas distal segments, particularly the colon, are dominated by *Bacteroidaceae, Lachnospiraceae*, and *Ruminococcaceae*. A transversal stratification also exists: species such as *Bacteroidaceae, Enterococcaceae*, and *Lactobacillaceae* prevail in the lumen, while *Lachnospiraceae* and *Ruminococcaceae* are concentrated near the mucosal surface [230].

**Figure 4:**
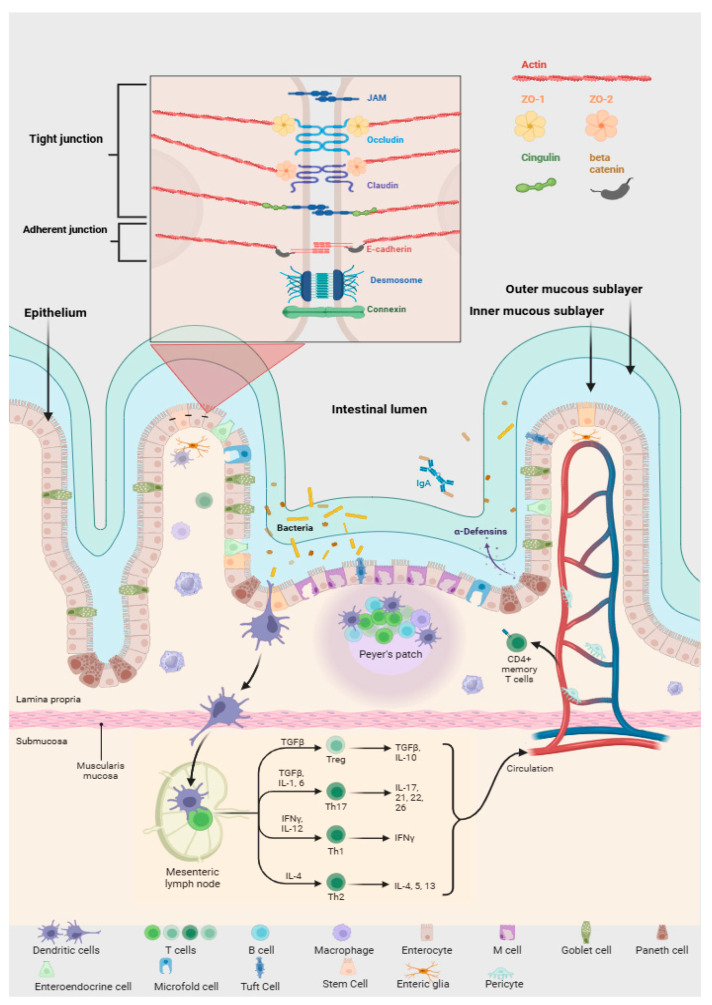
The intestinal barrier in disorders of the CNS: JAM, junctional adhesion molecule; ZO-1, zonula occludens-1; ZO-2, zonula occludens-2; IgA, immunoglobulin A; CD4^+^, cluster of differentiation 4 positive T lymphocytes; TGF-β, transforming growth factor beta; IL, interleukin; Th1, T helper 1 cells; Th17, T helper 17 cells; Treg, regulatory T cells.

The intestinal epithelium represents the second major structural layer of the intestinal wall and is composed of a single layer of specialized epithelial cells, including enterocytes, goblet cells, Paneth cells, enteroendocrine cells, and microfold cells. Enterocytes constitute the predominant cell type and are primarily responsible for the absorption of nutrients. Goblet cells, accounting for approximately 10% of the epithelial population, secrete mucus that forms a protective coating against digestive enzymes and mechanical damage [225]. Paneth cells, located at the base of intestinal crypts, contain granules rich in AMPs that provide a continuous defense against microbial invasion. Their secretion can markedly increase during infection or stress, enhancing the antimicrobial capacity of the mucosa [231]. The epithelial cells beneath the mucus are interconnected by TJ proteins that tightly regulate paracellular permeability and the exchange of solutes. These junctions include transmembrane proteins, occludin, claudins, junctional adhesion molecules, and tricellulin, and cytoplasmic adaptor proteins such as zonula occludens (ZO-1, ZO-2, and ZO-3), which link these structures to the actin cytoskeleton (Figure 4). The composition and activity of the intestinal microbiota directly influence the expression and organization of TJ proteins, emphasizing the strong interconnection between microbial balance and epithelial integrity [228]. Enteroendocrine cells, dispersed among enterocytes, synthesize and release hormones that regulate intestinal motility, digestive enzyme secretion, insulin release, satiety, and immune modulation [232]. Microfold cells facilitate antigen sampling by transporting microorganisms and luminal antigens across the epithelium to immune cells in the underlying lamina propria, thereby orchestrating local immune responses. Beneath the epithelial layer lies the lamina propria, which contains the enteric immune system composed mainly of leukocytes, with macrophages and DCs as the dominant populations. Intestinal macrophages, located in close proximity to the microbiota, continuously sample luminal microorganisms to preserve immune balance. Under normal conditions, their interactions with commensals occur without triggering inflammation due to constitutive IL-10 signaling [225]. During immune activation, infiltrating monocytes can differentiate into proinflammatory macrophages that produce cytokines such as IL-1β, IL-6, and TNF, as well as ROS, amplifying inflammatory cascades. Inflammatory states can also promote the release of proteases from both epithelial and immune cells into the lamina propria, leading to degradation of epithelial structures and impaired barrier function. Such damage contributes to acute or chronic intestinal inflammation [233].

A further protective component is the gut vascular barrier (GVB), formed by endothelial cells in association with enteric glial cells and pericytes. This barrier regulates the selective passage of molecules from the gut lumen to the bloodstream. Experimental evidence demonstrates that fluorescein isothiocyanate (FITC)-dextran of 4 kDa crosses the GVB freely, whereas 70 kDa FITC-dextran does not. However, infection with Salmonella enterica compromises GVB integrity, allowing larger molecules to penetrate. The GVB functions as a selective filter that prevents excessive antigen translocation and protects the systemic circulation from microbial components and intestinal bacteria [234]. 

Persistent weakening of these structural and microbial defenses promotes paracellular leakage of antigens, toxins, and microorganisms into the circulation. Selective permeability represents one of the most essential functional properties of the intestinal wall. It allows the controlled passage of nutrients, ions, and water while restricting the penetration of pathogens and toxic molecules. Both endogenous factors, such as inflammation, and exogenous influences, including diet, xenobiotics, or pharmacological agents, can increase intestinal permeability and compromise barrier integrity [235]. Oxidative stress and inflammation further drive neurodegeneration, with aging-associated loss of antioxidant defenses fostering a chronic pro-inflammatory state often referred to as inflamm-aging [236,237].

Beyond barrier function, the GBA includes direct neural routes (VN, ENS) and the HPA axis. Microbiota-derived molecules influence neurotransmitters (dopamine, 5-HT, GABA, noradrenaline) and hormones (GLP-1, PYY), affecting brain function through neural or systemic pathways. Chronic stress and elevated IL-6 overactivate the HPA axis, raise cortisol, and exert neurotoxic effects [220,221,222]. Microbiota also engage immune signaling through the inflammasome, IFN-I, and NF-κB pathways. Cytokines such as IL-1β, IL-6, and IL-18 are linked to depression, while lactic acid bacteria stimulate IFN-I via TLR3 on intestinal DCs. NF-κB overactivation in gut and hippocampus correlates with amnesia, reversible after restoring microbial diversity [37].

## 8. Alzheimer’s Disease (AD)

AD, the most common neurodegenerative disorder, is associated with mutations in the APP and presenilin 1 (PSEN1) genes [238,239]. Its pathology is defined by amyloid-β (Aβ) plaques and tau neurofibrillary tangles, leading to cholinergic neuronal loss in the basal forebrain and other CNS regions. Aβ plaques result from abnormal APP cleavage by β- and γ-secretases and impaired clearance [108].

The ENS, largely cholinergic, also expresses APP. Pathological analysis of AD patients shows preserved ENS structure, suggesting compensatory neurogenesis [240]. In transgenic AD mouse models, intestinal changes exacerbate CNS pathology: APP/PS1 mice show neurotransmitter alterations in the ENS without neuronal loss, while intestinal inflammation in App^NL-G-F^ mice accelerates CNS amyloidosis, although direct evidence in humans remains limited [225,226].

Gut dysbiosis in AD is linked to disrupted neurotransmitter balance (glutamate, acetylcholine, dopamine, GABA, 5-HT, norepinephrine) [241,242]. Taxa such as *Escherichia/Shigella*, *Bacteroides*, and *Ruminococcus* increase, while *Eubacterium rectale*, *Bifidobacterium*, and *Dialister* decrease [243]. Reduced diversity, with lower *Lactobacillus*, *Bacteroides*, and *Prevotella* and higher *Ruminococcus*, *Atopobium*, and *Enterobacteriaceae*, correlates with frailty [242]. Overall, AD patients often exhibit more *Bacteroidota* and fewer *Bacillota*, *Proteobacteria*, and *Actinobacteria.* Similar shifts are seen in SAPM8 and other AD mouse models, with increased *Lachnospiraceae*, *Alistipes*, *Proteobacteria*, and *Erysipelotrichaceae* [243]. Antibiotic-induced microbiota perturbations also modify neuroinflammation and amyloidosis [244] (Table 2).

Metabolomic studies in mild cognitive impairment (aMCI) and AD highlight altered tryptophan metabolism: reduced 5-HTP correlates with cognitive decline, while increased aryl AhR ligands inversely associate with cognition. *Ruminococcus* abundance negatively correlates with indole-3-pyruvate [245,246,247,248,249,250,251,252,253,254]. AD models also show reduced butyric and isobutyric acids, paralleling brain metabolite deficits. Experimentally, Aβ hippocampal injection in rats shifts microbiota toward pro-inflammatory species, while fructooligosaccharide supplementation increases *Lactobacillus*, *Bifidobacterium*, and neurotransmitter-producing taxa, elevates acetylcholine, dopamine, 5-HT, and norepinephrine in the brain, and improves cognition [255].

FMT has shown promise. In AD mouse models, FMT reduces Aβ plaques, tau tangles, and cognitive impairment. A clinical case study further reported rapid improvements in memory and mood following FMT from a healthy donor, suggesting microbiota modulation may be a promising therapeutic avenue for AD [230].

**Figure 5:**
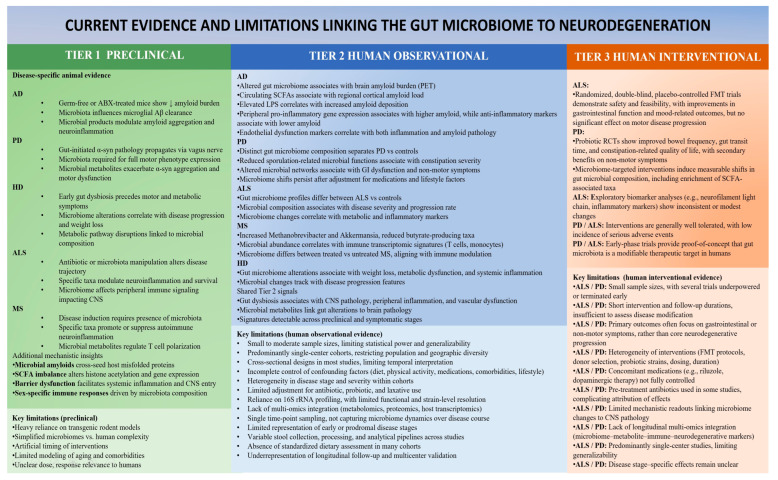
Current Human and Preclinical Evidence on Gut Microbiome-Neurodegeneration Interactions. AD, Alzheimer’s disease; PD, Parkinson’s disease; ALS, amyotrophic lateral sclerosis; MS, multiple sclerosis; HD, Huntington’s disease; DLB, dementia with Lewy bodies.

Recent evidence suggests a microbial contribution to AD pathogenesis, with some proposing a microbial origin [247]. Aβ may act as an antimicrobial peptide, and several bacteria produce amyloid-like proteins that exacerbate neurodegeneration, as observed in PD models [248]. Human cross-sectional studies show increased *Escherichia with Shigella* and reduced *Eubacterium rectale* in AD patients, correlating with higher systemic pro-inflammatory cytokines and brain amyloidosis, although larger longitudinal studies are needed (Figure 5) [255,256,257,258,259].

In transgenic mouse models, microbiota depletion reduces Aβ deposition and neuroinflammation. Germ-free animals show fewer plaques, while long-term antibiotic treatment lowers glial activation and insoluble Aβ accumulation [228,233,234]. Gut-derived molecules further contribute to pathology: LS is elevated two- to threefold in AD brains, correlates with cognitive decline [250], and induces memory loss and anxiety in mice [251]; PGN crosses the BBB to alter gene transcription [252]; bacterial extracellular DNA promotes tau aggregation and Aβ misfolding [253]; and microbial amyloids such as FapC accelerate Aβ fibrillization and cognitive impairment in zebrafish AD models [260,261,262,263,264,265,266,267].

Microbial metabolites further modulate AD pathology. SCFAs can inhibit Aβ aggregation in vitro, but are reduced in AD models, correlating with gut structural changes and bacterial amyloid deposition [239,240]. Butyrate protects neurons from Aβ toxicity [135], while bile acids, often altered in AD, may serve as diagnostic biomarkers [256]. Trimethylamine N-oxide (TMAO) promotes Aβ aggregation, accelerates hippocampal senescence, and worsens cognition, while lowering TMAO improves memory [242,243]. Microbiota-derived GABA regulates cortical excitability, and restoring inhibitory tone improves cognition in AD mice [259].

Aberrant Wnt/β-catenin signaling contributes to Aβ and tau accumulation [260]. Microbiota-derived amyloids and LPS promote neuroinflammation and Aβ deposition via intestinal barrier disruption, TLR4/TNF-α activation, and systemic inflammation [261]. Dysbiosis in AD typically includes reduced *Bifidobacterium* and *Lactobacillus* with increased *E. coli* and *Helicobacter pylori* [247,248]. FMT from healthy donors to AD models improves Aβ/tau pathology, reduces glial activation, and enhances cognition [249,250,251].

AD is linked to osteoporosis through mechanisms such as skeletal amyloid deposition, which activates NF-κB receptor signaling and osteoclast activity. Patients show reduced hip bone density and nearly double the risk of fracture. Vitamin D deficiency or reduced vitamin D-binding protein may impair amyloid clearance, while estrogen deficiency in postmenopausal women contributes to vulnerability in both brain aging and bone metabolism. AD pathology extends beyond the brain. Neurodegeneration and phosphorylated tau have been found in the spinal cord, with severity decreasing from cervical to sacral regions. The absence of peripheral ganglia involvement suggests a CNS originating, top-down spread [266,267]. Oral pathogens such as *Porphyromonas gingivalis* have been detected in AD brains, where gingipains promote Aβ_1–42_ accumulation and tau damage [268].

Other microbes, including HSV-1, fungi, *Chlamydia pneumoniae*, and spirochaetes, have also been implicated, with HSV-1 elevating cholesterol 25-hydroxylase (CH25H), a regulator of Aβ production [269]. In AD models, microbiota alterations coincide with gut barrier breakdown: 5xFAD mice show tight/adherens junction loss and NLRP3 inflammasome activation [270], while Tg2576 mice develop vascular Aβ deposition in the intestine before cerebral plaques, linked to malabsorption and elevated cytokines such as IL-9, IP-10, and VEGF [271].

The gut microbiome exhibits a characteristic reduction in *Bacillota* and *Bifidobacterium* species, both known producers of SCFAs, alongside enrichment of proinflammatory *Proteobacteria* and *Bacteroides* taxa. This dysbiosis contributes to compromised intestinal barrier function and elevated systemic levels of inflammatory mediators such as IL-6 and TNF-α. Bacterial metabolites and amyloid-like peptides can cross the intestinal and BBB, promoting microglial activation and amyloid β deposition. In addition, alterations in the gut microbiota modulate tryptophan metabolism and reduce production of indole derivatives, which normally exert neuroprotective and anti-inflammatory effects [272]. The increased *Bacteroides* and reduced *Ruminococcus* and *Bifidobacteria* may disrupt the gut barrier, allowing LPS, SCFAs, and amyloid proteins to influence the brain. *Bacteroides fragilis* produces fragilysin, a toxin that damages epithelial junctions and activates NF-κB signaling, processes associated with amyloid plaque formation [80,81,82].

A 2024 systematic review and meta-analysis reports consistent enrichment of pro-inflammatory Gram-negative bacteria and depletion of commensals that sustain butyrate production, aligning with elevated LPS and low SCFAs in AD cohorts. Mechanistically, these shifts associate with higher systemic cytokines and amyloid/tau pathology [273,274].

Elderly AD patients often show reduced butyrate-producing bacteria, important for anti-inflammatory activity and immune balance, alongside enrichment of pro-inflammatory taxa. Therapeutically, shifting microbiota composition toward anti-inflammatory metabolism may support gut–brain homeostasis and slow disease progression [246].

## 9. Parkinson’s Disease (PD)

PD, the second most common neurodegenerative disorder, affects 1–2% of people over 65 and is marked by progressive loss of dopaminergic neurons in the substantia nigra pars compacta (SNpc), leading to striatal dopamine depletion [275,276,277,278]. Clinically, PD presents with motor and behavioral symptoms as well as GI dysfunction [259,260]. Definitive diagnosis is postmortem, by identifying Lewy bodies (LBs), aggregated α-synuclein (α-SYN), in brain tissue [260,261]. Importantly, LB pathology is also observed in the ENS, suggesting peripheral involvement [259].

Braak’s staging system describes disease progression from the lower brainstem (stage 1) through the midbrain (stage 3) to widespread cortical involvement (stages 5–6) [279]. Early LB presence in ENS and brainstem led to the hypothesis that PD pathology may originate in the gut and spread to the CNS via the VN, consistent with prodromal GI symptoms and the apparent protective effect of vagotomy [54]. Recent preclinical findings further support the ‘gut-origin’ hypothesis, although direct evidence for this mechanism in humans remains limited [54,264].

Experimental evidence supports gut–brain transmission. In toxin-based models, intestinal exposure induces α-SYN misfolding and dopaminergic neuron loss in the brain, while inoculating the gut with PD patient brain lysate, recombinant α-SYN, or preformed fibrils (PFFs) drives CNS pathology in a prion-like, templated manner. This transmission requires endogenous α-SYN and is prevented by vagotomy [265,266]. In the 6-hydroxydopamine (6-OHDA) lesion model, intestinal motility deficits correlate with loss of neuronal NOS1 and increased tyrosine hydroxylase expression. Together, these findings highlight the bidirectional nature of gut–brain interactions in PD pathogenesis [280,281,282,283].

Despite persistent dysfunction, the ENS structure is often preserved in PD patients and gut-inoculated models [268,269]. Genome-wide association studies (GWAS) have identified ~90 PD-associated loci (e.g., SNCA, LRRK2, PINK1, PARKIN), accounting for 16–36% of heritable risk. In A53T-transgenic mice, mutant SNCA induces severe ENS dysfunction before CNS pathology, possibly due to more efficient neuronal clearance in young brains [284,285,286]. Clinically, PD is characterized not only by motor impairment but also by GI symptoms such as constipation, appetite and weight loss, dysphagia, sialorrhea, and gastroesophageal reflux (GERD). Aggregated α-SYN is found in submucosal and myenteric plexuses before brain involvement, supporting a gut-to-brain prion-like spread [269,287,288,289,290].

Microbiota analyses show an ~80% reduction in Prevotellaceae in PD patients [271,272] (Table 2). PD progression may follow different trajectories: a bottom-up gut-to-brain pattern consistent with Braak staging, often associated with REM sleep behavior disorder (RBD), or a top-down brain-to-gut pathway. RBD may serve as a clinical biomarker for subtype and progression [273,275].

Before motor onset, PD involves gut dysbiosis, ENS-immune uncoupling, reduced host defense, and colonic inflammation [291]. Characteristic microbial changes include increased *Akkermansia, Catabacter, Lactobacillus, Bifidobacterium, Ruminococcaceae, Verrucomicrobiaceae*, and *Christensenellaceae*, alongside reduced *Roseburia*, *Faecalibacterium*, *Lachnospiraceae ND3007*, *Prevotellaceae*, *Blautia*, *Coprococcus*, and *Lachnospira* [277,278]. Notably, *Enterococcus faecalis* interacts with L-DOPA metabolism in PD patients. In mouse models, berberine enhanced L-DOPA biosynthesis by increasing tyrosine hydroxylase activity in *E. faecalis*, enabling L-DOPA to cross the BBB, convert to dopamine, and improve symptoms [292,293,294].

FMT from healthy donors has shown benefits in PD patients, improving motor function, sleep, mood, constipation, and quality of life [280,281]. In PD models, FMT restored microbial balance, increased striatal dopamine and 5-HT, and reduced glial activation in the substantia nigra, supporting neuroprotection [295,296,297].

Microbial metabolites such as propionate also alleviate motor deficits and enhance dopaminergic neuron survival, likely via ENS receptor signaling. OP and osteopenia are frequent in PD, with lower serum 25(OH)D compared to AD patients and healthy controls [298]. Contributing mechanisms include α-SYN aggregation in bone cells and gut microbiota-driven changes in inflammation and hormone regulation, leading to bone loss. Women with PD have ~2.1% lower bone mineral density (BMD) than non-PD women. These observations support the proposed “brain–gut–bone axis,” linking α-Syn pathology, autonomic dysfunction, intestinal permeability, and microbial metabolites to skeletal decline [18,284,299,300].

Non-vagal transmission pathways also appear relevant in PD. α-Syn aggregates have been identified in celiac ganglia and spinal intermediolateral nuclei, consistent with spread via glymphatic, immune, endothelial, or cerebrospinal fluid routes [275,285]. Microbial molecules are emerging as PD biomarkers. LPS induces microglial activation and dopaminergic neuron loss via mitochondrial dysfunction [301], while PGNs engage host PRRs and increase PD risk [287,288]. Bacterial amyloids such as Fap (Pseudomonas) and Curli (Enterobacteriaceae) promote α-Syn aggregation and motor deficits [302,303,304]. Chronic macrophage activation in the submucosa and muscularis can damage ENS neurons and glia, propagating pathology to the CNS through peripheral nerves and humoral loops, ultimately contributing to nigrostriatal dopaminergic loss [291].

A major limitation in the current literature concerns the interpretation of short-chain fatty acid (SCFA) measurements without adequate consideration of biological compartmentalization. Meta-analytical evidence consistently shows reduced fecal levels of acetate, propionate, and butyrate alongside increased plasma concentrations of butyric, propionic, and isobutyric acids in Parkinson’s disease. These opposing trends challenge simplistic interpretations of “SCFA deficiency” and indicate that fecal and circulating SCFAs represent distinct physiological pools rather than interchangeable readouts of microbial activity. Consequently, conclusions drawn from stool measurements alone risk misrepresenting systemic exposure and downstream biological effects [194].

The observed stool-plasma divergence likely reflects altered intestinal handling rather than uniform changes in SCFA production. Because the majority of luminal SCFAs are absorbed by colonocytes, only a small fraction remains detectable in feces. Reduced fecal SCFAs may therefore arise from enhanced epithelial uptake, prolonged intestinal transit, or altered transport dynamics, rather than decreased microbial fermentation per se. This distinction is frequently overlooked, leading to overinterpretation of fecal SCFAs as direct indicators of microbial output [196].

Intestinal barrier dysfunction represents a critical, yet inconsistently integrated, confounder. Increased gut permeability has been documented in Parkinson’s disease, as evidenced by elevated fecal zonulin and calprotectin levels, and supported by experimental models showing early tight junction disruption and intestinal inflammation [193]. Such barrier impairment facilitates excessive translocation of luminal metabolites into the circulation, providing a plausible explanation for elevated plasma SCFAs despite reduced fecal concentrations. Failure to account for barrier integrity substantially limits causal inference regarding SCFA alterations and their systemic effects [198].

Dietary intake of fermentable substrates varies widely and strongly influences SCFA production yet was inconsistently reported or adjusted for across studies [196]. Gastrointestinal dysfunction, especially constipation, affects up to 80% of patients and profoundly alters microbial composition, intestinal transit time, and SCFA absorption [195]. Although SCFAs can cross the BBB, data on cerebrospinal fluid and brain tissue concentrations remain scarce and inconsistent, precluding robust conclusions regarding central exposure. Moreover, SCFAs undergo dynamic metabolic processing by colonocytes and the liver before reaching systemic circulation, generating steep concentration gradients across compartments that are rarely considered in study design [196].

In PD, patients show reduced fecal SCFAs and lower abundance of SCFA-producing taxa such as *Roseburia* and *Faecalibacterium*, while plasma SCFA levels paradoxically increase with disease severity and treatment [293]. TMAO has been detected in cerebrospinal fluid, with high plasma levels linked to terminal PD and low levels associated with early-stage risk, although its biomarker potential remains unclear [230,279]. Hydrogen-producing bacteria are also reduced in PD [295], and molecular hydrogen exhibits neuroprotective properties: hydrogen-rich water decreases inflammation and apoptosis in healthy adults, protects dopaminergic neurons in mouse models, and improves motor symptoms in patients [296,297].

Gut dysbiosis contributes to α-Syn misfolding in the ENS. Reduced *Prevotellaceae* impairs SCFA, thiamine, and folate production, increasing gut permeability, systemic LPS, and colonic α-Syn aggregation [298]. Higher *Enterobacteriaceae* abundance correlates with postural instability, gait impairment, and elevated serum LPS-binding protein [284,285]. Loss of TJ proteins (e.g., occludin) [301], upregulated TLR4, and increased proinflammatory cytokines (TNF-α, IL-1β, IL-6) in colonic tissue promote systemic inflammation and BBB compromise [302,303]. TLR4-knockout mice show reduced inflammation, motor impairment, and neurodegeneration in rotenone-induced PD, confirming its role in pathology. Elevated *Ralstonia*, *Enterococcus*, and *Proteobacteria* also contribute to inflammation, whereas butyrate producers are diminished [304].

Specific pathogens may trigger PD. *Helicobacter pylori* infection increases risk, and SIBO worsens motor dysfunction [305]. *Porphyromonas gingivalis* releases gingipain (RgpA) and LPS, promoting abnormal clotting, dopaminergic loss, gut permeability, and microglial activation, particularly in LRRK2-associated PD [306].

Although quantitative syntheses provide valuable confirmation of reproducible microbial signatures in PD, several methodological limitations temper the strength of these conclusions. The reported mean differences for *Prevotellaceae*, *Faecalibacterium*, and *Lachnospiraceae* (MD ≈ −0.3 to −0.4) and for *Bifidobacteriaceae*, *Ruminococcaceae*, and *Verrucomicrobiaceae* (MD ≈ +0.4–0.6) suggest consistent taxon-level alterations across cohorts. These effect sizes remain moderate and were derived from highly heterogeneous case–control studies differing in sequencing platforms, sample handling, dietary background, medication use, and regional microbiota baselines [281].

Furthermore, the broader meta-analysis including 24 studies (*n* = 1469 PD; *n* = 1289 controls) revealed only a small, statistically marginal reduction in α-diversity (SMD = −0.30; 95% CI = −0.51 to −0.08; I^2^ = 22%). This indicates that compositional, rather than global diversity, changes dominate PD dysbiosis. The limited magnitude of diversity effects questions their diagnostic specificity and underscores the influence of confounding factors such as constipation, dietary fiber intake, and dopaminergic therapy variables often insufficiently controlled across studies [307].

Importantly, the analytical focus on relative abundance without parallel quantification of microbial metabolites (e.g., SCFAs, bile acids) or host immune markers restricts mechanistic interpretation. Future meta-analyses integrating metagenomic, metabolomic, and clinical phenotyping data are necessary to distinguish disease-specific microbial alterations from epiphenomena of PD-associated lifestyle or pharmacological factors. Microbiota-targeted strategies are under investigation. *Bacillus* spp. convert L-tyrosine to L-DOPA [308], while *Lactobacillus* spp. improve bowel function in PD. Flavanol-derived phenolic acids from gut microbes interfere with α-Syn misfolding and toxicity in humanized mice, *Drosophila*, and in vitro systems [309].

The gut microbiota also modulates drug metabolism: certain bacteria degrade levodopa and carbidopa, influencing efficacy and toxicity [310,311,312,313,314]. Diets rich in SCFA precursors, such as vegetarian diets, may enhance UDP-glucuronosyltransferase activity and reduce levodopa requirements. Gut microbiota profiling, extracellular vesicle analysis, and FMT are being explored for diagnosis and therapy, though current evidence does not yet validate microbiome-derived biomarkers in PD [295,296,315,316,317,318,319,320].

Several studies have reported a consistent decrease in beneficial bacteria such as *Prevotella* and *Roseburia* and an increase in *Enterobacteriaceae*, *Desulfovibrio*, and *Clostridium* species. These alterations are associated with the production of LPS and hydrogen sulfide, which can damage the intestinal epithelium and trigger α-SYN aggregation in the ENS. Increased intestinal permeability facilitates the translocation of bacterial endotoxins that activate microglia in the vagal nuclei and substantia nigra. Chronic exposure to LPS induces local inflammation and oxidative stress, accelerating dopaminergic neuron loss [292]. Disruption of vagal afferents to the NTS and altered noradrenergic signaling from the locus coeruleus further drive disease progression [321,322].

Methodological heterogeneity further undermines comparability across studies. SCFA quantification has relied on diverse analytical platforms, including GC-MS and LC-MS/MS, which differ markedly in derivatization requirements, sensitivity, and pre-analytical stability [323,324]. Variations in sample storage, solvent use, lyophilization, fecal hydration, and delays between collection and analysis introduce additional bias. These inconsistencies complicate cross-study synthesis and likely contribute to the high heterogeneity observed in meta-analyses, particularly for fecal butyrate [325].

Disease severity, cognitive status, and medication use have also been associated with SCFA concentrations but were incompletely documented in several cohorts, further limiting interpretability. Beyond plasma, elevated SCFA levels have been reported in saliva and urine, suggesting systemic redistribution rather than localized gut changes [326,327,328,329].

In PD, *Helicobacter pylori* infection worsens motor symptoms and reduces levodopa absorption, while eradication improves outcomes. The VN has also been implicated, with vagotomy linked to lower PD risk, highlighting its role in disease progression [44,78,79,330].

## 10. Amyotrophic Lateral Sclerosis (ALS)

ALS is a progressive neurodegenerative disorder involving degeneration of upper motor neurons in the cortex and lower motor neurons in the brainstem and spinal cord [331]. Its main pathological feature is cytoplasmic inclusions of TDP-43 in motor neurons. GI dysfunction is common, including delayed gastric and colonic motility. In mutant TDP-43 (TARDBP) mice, selective loss of inhibitory NOS1^+^ myenteric neurons caused intestinal obstruction and sudden death, with RET receptor tyrosine kinase signaling required for neuronal preservation [332].

Therapeutically, microbiome-based approaches remain limited. Inosine, a postbiotic metabolite, was tested in a small clinical trial (3 g/day for 20 weeks) to raise serum urate, a biomarker associated with longer survival. While serum urate increased, no clinical benefit was observed, and renal complications occurred in some patients. To date, no probiotic-based therapies have been tested in ALS or other motor neuron diseases [333].

ALS pathology involves multiple molecular mechanisms, including neuroinflammation, impaired RNA metabolism, oxidative stress, mitochondrial dysfunction, cytoskeletal disruption, altered exon splicing, impaired nucleocytoplasmic and axonal transport, toxic protein aggregate accumulation, autophagy disturbances, and glutamate-induced excitotoxicity [334]. Mutations in approximately 30 genes have been identified as causative. Only riluzole and edaravone are FDA-approved treatments, both providing modest slowing of disease progression. Elevated Wnt/β-catenin signaling in motor neurons, astrocytes, microglia, and oligodendrocytes contributes to neuromuscular junction impairment, with acetylcholine receptor clustering playing a critical role in junction formation [335].

Gut microbiota composition differences in ALS include reduced butyrate-producing bacteria important for gut integrity and inflammation control, while in Sod1 transgenic mice, Akkermansia muciniphila improved symptoms and *Ruminococcus* and *Desulfovibrio* worsened them [336]. ALS-related musculoskeletal decline, partly from physical inactivity and malnutrition, is linked to decreased bone mineral density, increased fracture risk, and trabecular bone deterioration. Dysbiosis-driven inflammation may contribute to both neurological and bone pathology, suggesting a potential therapeutic target in the brain–gut–bone axis [337].

ALS is characterized by progressive degeneration of motor neurons and is increasingly linked to alterations in the gut microbiome. Several studies report reduced abundance of *Akkermansia muciniphila*, *Butyrivibrio fibrisolvens*, and *Ruminococcus* species, microbes involved in the production of butyrate and nicotinamide, both crucial for neuronal energy metabolism and anti-inflammatory protection. Loss of these beneficial taxa results in metabolic stress, impaired intestinal barrier integrity, and chronic systemic inflammation that extends to the CNS. In mouse models, oral supplementation with *Akkermansia muciniphila* restored nicotinamide levels, reduced neuroinflammation, and improved motor performance, suggesting a causal microbiota-neuron relationship [338,339].

Clinical studies report that GI symptoms can appear before neurological manifestations in ALS, with fecal analysis showing reduced intestinal microbiota diversity compared to healthy controls [340]. Alterations include a decreased *Bacillota/Bacteroidota* ratio and lower abundance of *Anaerostipes*, *Oscillibacter*, and *Lachnospiraceae* (Table 2). This dysbiosis may compromise the intestinal epithelial barrier, promote inflammatory responses, and impair bowel motility. It has been hypothesized that barrier dysfunction allows translocation of toxins such as LPS into the bloodstream, triggering innate immune activation relevant to ALS pathogenesis [14].

Additionally, the gut microbiota-derived metabolite nicotinamide improves motor function and gene expression in ALS mouse models, while systemic and cerebrospinal fluid levels of nicotinamide are reduced in ALS patients [341].

## 11. Multiple Sclerosis (MS)

MS is a chronic, immune-mediated neurological disorder of the CNS characterized by axonal damage, demyelination, and the formation of inflammatory plaques in gray and white matter. Disruption of the BBB permits infiltration of immune cells, particularly myelin antigen-specific CD4^+^ and CD8^+^ T cells, which drive neuroinflammation and neurodegeneration [147]. CD4^+^ Th1 and Th17 cells play central roles: Th1 cells produce IFN-γ, IL-12, and TNF-α, inducing chronic inflammation and oxidative/nitrosative stress, while Th17 cells secrete IL-17, IL-21, and IL-22, sustaining disease activity. NK cells, microglia, and macrophages also contribute to the inflammatory cascade. MS arises from both genetic and environmental factors, with the gut microbiota emerging as a key environmental risk modulator of immune responses, BBB integrity, and autoimmune demyelination [342,343,344,345].

Cross-sectional studies, particularly in children within two years of disease onset, show discrete taxonomic shifts rather than broad α- or β-diversity changes [309,310,311]. FMT from MS patients into experimental EAE models increases disease severity, while germ-free mice are resistant to EAE. Colonization with segmented filamentous bacteria restores susceptibility via Th17 activation, whereas IL-10-producing CD4^+^ T cells mediate protective microbiota-driven effects. Two small human trials reported symptomatic improvement after FMT [346,347,348,349,350,351].

The microbiota also regulates myelin production in the prefrontal cortex and maintains BBB integrity. Supplementation with SCFAs or colonization with SCFA-producing bacteria reverses barrier disruption in animal models [347]. Diet-induced microbial changes influence EAE onset and severity, and early clinical data suggest therapeutic potential: a multispecies probiotic (*Lactobacillus*, *Bifidobacterium*, *Streptococcus*) administered for two months reversed microbiota alterations and showed anti-inflammatory effects in MS patients (Table 2). While promising, larger trials are needed to confirm microbiota-targeted interventions as strategies to reduce relapse risk and symptom severity [331,332].

Recent evidence highlights the significant role of gut microbiota in MS pathogenesis, with both internal microbial imbalances and external environmental factors influencing disease progression. Microbiota profiling via 16S rRNA sequencing has revealed that untreated MS patients often exhibit increased *Bacillota*, *Euryarchaeota*, and *Akkermansia*, alongside decreased *Bacteroidota*, *Prevotella*, and *Clostridium*, the latter linked to reduced SCFA secretion [324]. External factors such as Epstein–Barr virus infection, smoking, obesity, and vitamin D deficiency can modify the gut microbiota and worsen MS risk or symptoms. Obese MS patients often show similar microbial shifts, with higher *Bacillota* and *Actinobacteria*, lower *Bacteroidota*, and reduced serum vitamin D_3_ [352,353,354].

In relapsing-remitting MS (RRMS), 16S rRNA studies revealed depletion of butyrate- and propionate-producing taxa, particularly *Eubacterium rectale* and *Megamonas funiformis*, while metagenomic and metabolomic analyses confirmed reduced SCFA availability [335,336]. SCFAs promote regulatory T cell induction and CNS remyelination [355]. Oral propionate supplementation improves clinical parameters and increases Tregs in MS patients, while animal studies confirm the therapeutic effects of both butyrate and propionate [356].

While recent quantitative syntheses provide valuable standardization across geographically and methodologically heterogeneous studies, their findings highlight the modest and taxon-specific nature of gut microbial alterations in MS. A 2024 meta-analysis reported no significant α- or β-diversity differences between MS patients and healthy controls, but reproducible directional trends emerged, namely an increase in *Actinomyces* and a reduction in *Faecalibacterium*, both with moderate effect sizes (Cohen’s d ≈ 0.5). These patterns suggest that MS-associated dysbiosis manifests through targeted shifts in immune-modulatory taxa rather than global microbial depletion [357].

However, these statistical associations remain moderate and are drawn from datasets characterized by substantial interstudy variability in sequencing depth, stool sampling timing relative to relapse/remission phases, and prior immunomodulatory treatment exposure. Many studies rely on relative abundance rather than absolute quantification, which may obscure clinically meaningful differences. The assumption of a d = 0.5 effect size borrowed from preliminary cohorts underscores the need for adequately powered, longitudinal, and mechanistically integrated designs [358,359].

Future meta-analytic efforts should integrate microbial taxonomic data with metabolomic profiles (e.g., SCFAs, bile acids) and host immune markers to delineate mechanistic pathways linking the gut microbiota to MS activity and progression. Without this systems-level integration, most current findings remain correlative rather than explanatory.

Comparative metagenomic and metabolomic profiling of RRMS and secondary progressive MS (SPMS) demonstrated disease stage-specific differences. SPMS patients showed increased microbial genes linked to DNA mismatch repair and higher oxidative stress signatures, suggesting oxidative stress-driven DNA repair activity in the microbiome may contribute to progression [338,339]. MS is an immune-mediated demyelinating disease in which gut microbiota composition strongly influences peripheral and central immune homeostasis. Characteristic changes include elevated *Akkermansia muciniphila* and *Methanobrevibacter smithii* and decreased abundance of *Faecalibacterium prausnitzii* and *Bacteroides fragilis*, species involved in Treg differentiation and SCFA synthesis. These shifts promote a pro-inflammatory milieu by enhancing Th17 activity and reducing Treg function. In EAE models, colonization with *Akkermansia* and *Methanobrevibacter* exacerbates demyelination, whereas *Bacteroides fragilis* polysaccharide A exerts protective IL-10-mediated tolerance [343,360].

Clinical and pilot trials indicate that probiotics can beneficially modulate inflammation and outcomes in MS [360,361]. Randomized controlled trials with *Lactobacillus* and *Bifidobacterium* mixtures improved depression, anxiety, metabolic parameters, and downregulated pro-inflammatory cytokines [362]. Six months of the commercial probiotic Protexin reduced pain and fatigue, increased BDNF, and elevated nitric oxide with potential neuroprotective effects [331,343,344].

## 12. Huntington Disease (HD)

HD is a rare, autosomal dominant neurodegenerative disorder caused by CAG trinucleotide repeat expansion in the Huntingtin (HTT) gene [363]. Mutant Huntington gene (HTT) leads to fragmented protein accumulation and progressive neuronal loss, initially affecting striatal GABAergic medium spiny neurons projecting to the substantia nigra and globus pallidus, and later causing widespread brain atrophy. Clinical features typically manifest between ages 35–44 and include cognitive decline, psychiatric symptoms, and progressive motor dysfunction, advancing to dysphagia, weight loss, and aspiration-related complications [346,347]. Non-neurological manifestations such as weight loss have been linked to intestinal dysfunction and ENS neurodegeneration, documented in both HD patients and R6/2-transgenic mouse models [364,365,366]. In mice, this is associated with reduced neuropeptide expression, malabsorption, and hypothalamic neurodegeneration that impairs drinking and swallowing, further promoting weight loss [331].

Patients with HD exhibit reduced microbial diversity, with decreased levels of *Eubacterium rectale* and *Bacteroides fragilis* and enrichment of proinflammatory *Clostridium* cluster XIVa species. These microbial imbalances correlate with increased intestinal and systemic cytokines (IL-6, TNF-α, IL-1β), which may enhance BBB permeability and promote neuroinflammatory signaling in the striatum. Altered microbial metabolism affects the production of tryptophan and kynurenine pathway metabolites, contributing to excitotoxicity and mitochondrial dysfunction [367,368,369].

Multi-omics studies suggest that gut microbiota may influence HD pathogenesis by modulating plasma metabolites. A distinct serum metabolic profile, partly derived from microbial activity, has been observed in both presymptomatic and early symptomatic patients, offering potential biomarkers for disease onset, progression, and phenotypic variability [20,349].

Altered gut microbiota composition has been linked to HD (Table 2) onset and progression, with possible sex-specific differences. In mouse models, males displayed increased *Bacteroidales* and *Lactobacillales* with reduced *Clostridiales*, while females exhibited elevated *Coriobacteriales*, *Erysipelotrichales*, *Bacteroidales*, and *Burkholderiales*, along with decreased *Clostridiales*. Male HD mice also showed greater microbial diversity than both female and wild-type counterparts [350,351]. Microbiota deficiency in mice reduced myelin-related proteins and mature oligodendrocytes in the prefrontal cortex, impairing callosal myelination and white matter plasticity, thereby worsening HD phenotypes. Specific microbial metabolites, including 5-HT, tyrosine, and hydroxyphenylacetic acids, may disrupt GBA signaling, while indole-3-propionic acid has been associated with increased intestinal permeability [370,371].

Although human meta-analysis is limited, convergent multi-omics and interventional data show reduced microbial diversity with depletion of *Eubacterium rectale*/*Bacteroides fragilis* and enrichment of pro-inflammatory *Clostridium* cluster XIVa; these shifts correlate with higher IL-6/TNF-α/IL-1β and metabolic signatures (kynurenine pathway) linked to excitotoxicity [372,373,374,375].

## 13. Summary

Accumulating evidence underscores the pivotal role of the gut microbiota in regulating brain function and neurological health. Through the MGBA, intestinal microbes release metabolites, neuroactive compounds, and immunomodulatory molecules that modulate neurotransmitter synthesis, availability, and signaling. These mediators act directly via systemic circulation or indirectly through vagal and immune pathways, thereby influencing cognition, behavior, mood regulation, and vulnerability to disease.

Importantly, the gut microbiome is a modifiable system, opening avenues for therapeutic intervention. Dysbiosis has been consistently associated with disorders such as autism spectrum disorder, PD, depression, and schizophrenia, while strategies including dietary modulation, probiotics, targeted antibiotics, and FMT demonstrate therapeutic promise in restoring microbial and neurochemical homeostasis.

Future therapeutic approaches may increasingly focus on microbial metabolites and enzymes that regulate neurotransmitter pathways, while microbiome profiling could enable precision medicine by predicting drug responsiveness and optimizing treatment outcomes. Despite strong epidemiological and experimental evidence linking gut microbiome alterations to NDs such as Alzheimer’s and Parkinson’s, these interactions remain complex and bidirectional. Whether dysbiosis constitutes a causal factor or a downstream consequence of neurodegeneration remains unresolved.

Current evidence strongly supports the MGBA as a critical determinant of neurological health, integrating metabolic, immune, endocrine, and neural pathways into a unified framework of brain–gut communication. In AD, shifts in microbial composition, reductions in SCFAs producing taxa, and increases in proinflammatory bacteria and metabolites appear closely linked to disease pathology, potentially contributing to impaired barrier integrity, neuroinflammation, and synaptic dysfunction. Importantly, such alterations may precede overt clinical manifestations, highlighting their potential role as early biomarkers and targets for intervention. A fundamental question remains unresolved: whether dysbiosis acts as a causal driver in AD pathogenesis or emerges as a secondary consequence of ongoing neurodegeneration.

Future research must therefore adopt longitudinal and multi-omics strategies, integrating metagenomics, metabolomics, transcriptomics, and proteomics, to disentangle these complex interactions and clarify temporal dynamics. Equally important is the consideration of interindividual variability shaped by host genetics, environment, diet, and pharmacological exposures, which can profoundly influence microbiota composition and responsiveness to interventions. Addressing these variables may allow for the development of precision-based strategies, where microbiota modulation through diet, probiotics, prebiotics, FMT, or small-molecule metabolites, becomes part of personalized therapeutic regimens.

Ultimately, advancing our understanding of the MGBA in AD has the potential to transform current disease models, bridging the gap between molecular neuropathology and systemic influences, and paving the way for novel diagnostic and therapeutic approaches. By positioning the microbiome not merely as a bystander but as an active participant in neurodegenerative processes, future studies may unlock new opportunities to delay, mitigate, or even prevent the progression of AD.

A deeper understanding of the MGBA could fundamentally reshape perspectives on PD, providing new insights into its early mechanisms and progression. Clarifying how gut microbiota alterations contribute to disease onset may enable the identification of peripheral biomarkers within the ENS, potentially before the appearance of overt neurological symptoms. Establishing causal links in this relationship will be essential for redefining the pathophysiological framework of PD and identifying points where intervention may alter its trajectory.

In MS, evidence consistently demonstrates associations between gut microbiota dysregulation and CNS autoimmunity. Patients typically show increased *Akkermansia* and *Streptococcus*, alongside reductions in *Bacillota* and *Bacteroidota*, especially SCFA-producing taxa. These changes highlight the role of microbial metabolites in modulating immune tolerance and inflammation. Findings from EAE models reinforce the dual role of the microbiota, which can be either protective or pathogenic depending on composition. Gut-derived metabolites influence oligodendrocytes, astrocytes, and microglia, directly shaping demyelination and remyelination processes. Such insights suggest therapeutic potential through dietary modulation and microbiome based interventions.

In AD, the GMB exerts significant influence over neuroinflammation and cognitive decline. Dysbiosis and altered metabolite production contribute to pathology by modulating immune responses and neuronal activity. Preclinical studies show that antibiotic treatment or germ-free conditions reduce amyloid deposition and microglial activation, while promoting anti-inflammatory regulatory T cells in both periphery and CNS. These systemic immune shifts emphasize the capacity of the microbiome to shape central neuroinflammatory pathways. Such findings support microbiota targeted therapies as promising strategies to complement existing AD treatments and potentially slow disease progression.

Several bacterial taxa are more prevalent in patients, and disease-modifying therapies clearly alter microbiota composition. A key question is whether these microbial shifts precede disease onset or arise as secondary consequences of the disease or its treatment. Genetically determined microbiome traits may also influence disease course, emphasizing the need to clarify causality. Distinctions between the microbiome and microbiota further complicate interpretation, as does the question of whether gut microbial changes are as critical as those occurring in other body sites. Environmental exposures, including diet, stress, comorbidities, and especially antibiotic use, add further complexity. Given these confounding factors, findings must be interpreted cautiously, and future studies should employ robust approaches such as Mendelian randomization to minimize bias.

The GBA represents a network of biochemical pathways connecting gut microbiota to CNS function. Disruptions in microbial balance can impair endocrine, immune, and neural signaling, contributing to neuronal dysfunction and age related decline. Hallmarks of NDs include chronic inflammation, oxidative stress, impaired neurotransmitter balance, and altered metabolic signaling. Probiotics hold promise for restoring microbial equilibrium and supporting host-microbe interactions, yet mechanistic insights into their biochemical effects in neurodegeneration remain limited, requiring further study before broad therapeutic application.

Advances in microbiome research continue to reveal its role in health and disease, particularly in NDs such as AD and PD. Expanding knowledge in this area may enable earlier detection of disease through peripheral biomarkers and targeted preventive strategies. Dietary modulation appears to be an accessible tool for reshaping microbial balance with potential neurological benefits. At the same time, advanced methods such as fecal DNA sequencing provide noninvasive opportunities to assess microbial diversity and identify protective versus pathogenic taxa. In the long term, individual microbial signatures could serve as the basis for highly personalized interventions aimed at preserving brain health and mitigating neurodegeneration.

In the context of MS, this relationship appears particularly complex. Dysbiosis is consistently observed in patients, and the gut microbiota is recognized as a key modulator of the GBA with relevance to CNS pathology. No single microbial signature has been reliably associated with MS across studies. Divergent findings likely reflect differences in geography, sequencing platforms, disease stage, and therapeutic status. Since both microbiota composition and host genetic architecture are implicated in disease susceptibility, it has been proposed that part of the “missing heritability” in MS may reside in the interaction between genes and environmental exposures mediated through the microbiome. Clarifying these interactions could provide critical insights into disease risk, progression, and potential therapeutic targets.

Microbial diversity is a central concept in microbiome research and reflects the ecological complexity, stability, and resilience of microbial communities inhabiting the human body. Two major components are generally considered: alpha diversity, which describes the variability within a single sample, and beta diversity, which quantifies the compositional differences between samples. They provide a multidimensional view of microbial ecosystems, capturing both local richness and interindividual heterogeneity. High alpha diversity is often associated with ecological stability and host health, whereas reduced diversity may indicate dysbiosis or imbalance within the microbiota.

A broad range of quantitative indices has been developed to assess alpha diversity, each emphasizing distinct aspects of community structure. Richness estimators such as Observed Features, Chao1, and ACE measure the number of taxa present and attempt to infer those that may be undetected due to sequencing limitations. Dominance metrics, including the Simpson and Berger Parker indices, evaluate whether a few taxa dominate the community, providing information on the balance between abundant and rare species. Phylogenetic measures such as Faith’s Phylogenetic Diversity incorporate evolutionary relationships among taxa, offering a dimension that extends beyond mere abundance. Information theory based metrics such as Shannon’s entropy or Pielou’s evenness synthesize richness and evenness into a single numerical value, representing the uncertainty in predicting the identity of a randomly selected taxon. Each of these approaches captures different biological nuances, and their interpretation must take into account sequencing depth, normalization methods, and sample size.

The choice of diversity metric also has profound implications for statistical power and study design. Evidence from comparative studies has shown that the same dataset can yield very different effect sizes and significance levels depending on the metric applied. When comparing gut and oral microbial communities, effect size estimates varied more than threefold across alpha metrics, leading to large discrepancies in the number of samples required to achieve adequate statistical power. This demonstrates that microbiome studies may easily become underpowered or yield inconsistent conclusions purely because of metric selection. A survey of recent literature confirms that the Shannon index and Bray–Curtis dissimilarity remain the most frequently used measures, although they are not always the most sensitive for detecting subtle biological differences. Simulated and experimental datasets further indicate that Shannon’s index tends to be more responsive than Chao1 or Simpson when identifying group differences, while UniFrac and Bray–Curtis distances often provide superior discrimination in beta diversity analyses.

These observations highlight a persistent methodological challenge in microbiome research: the absence of standardized criteria for selecting and reporting diversity metrics. Without explicit justification and transparent reporting of effect size estimation and statistical assumptions, results can be difficult to compare or reproduce across studies. Consequently, consistent use of validated analytical pipelines, clear reporting of diversity measures, and explicit discussion of their limitations are essential for improving the robustness and interpretability of microbiome research. The integration of conceptually unified frameworks such as Hill numbers, although not yet widely adopted, may ultimately provide a more coherent and comparable approach to quantifying microbial diversity in biomedical investigations.

Over the past decade, research has revealed a strong connection between the microbiome and human health, raising fundamental questions about how microbial communities are established and the extent to which host genetics contribute. While studies in mice and humans, including twin cohorts, suggest that certain components of gut microbiota composition may be heritable, many human studies highlight the environment as the predominant determinant, with genetics exerting only a modest effect. Discrepancies across study designs, small sample sizes, and population-level variability have limited consensus on the precise genetic contribution.

Looking forward, advancing the field will require large scale, longitudinal, and multicenter studies that integrate multiomics approaches including metagenomics, transcriptomics, metabolomics, and host genomics, to disentangle causality within the GBA. Only by combining these layers can the intricate relationships between microbial dynamics, host genetics, and environmental influences be mapped with sufficient precision. Clinically, such efforts should inform the development of personalized interventions, where microbial signatures guide tailored strategies ranging from dietary modulation and targeted probiotics to next-generation therapies such as FMT and metabolite-based treatments. At the same time, careful consideration of potential risks and interindividual variability remains essential to ensure both efficacy and safety. Ultimately, unifying these perspectives may transform the microbiome from a correlational marker into a reliable biomarker and therapeutic target, opening the way for preventive and precision medicine approaches in neurodegenerative and psychiatric disorders.

## Figures and Tables

**Table 1:** Microbial genera and species producing neuroactive and metabolically active compounds: GABA, gamma-aminobutyric acid; 5-HT, serotonin (5-hydroxytryptamine); SCFA, short-chain fatty acid; TPH1, tryptophan hydroxylase 1; GPR41/43, G-protein-coupled receptors 41 and 43; ENS, enteric nervous system.

Microbial Genus/Species	Primary Bioactive Compound(s)	Mechanism/Effect	Evidence Type
*Faecalibacterium prausnitzii*	Butyrate (SCFA)	Anti-inflammatory; enhances epithelial barrier integrity and ENS homeostasis	Human & animal [74,75,76]
*Roseburia intestinalis*	Butyrate, propionate	Modulates motility and ENS signaling via GPR41/43	Human & animal [76]
*Eubacterium rectale*	Butyrate	Increases mucosal 5-HT and tight-junction protein expression	Human & animal [76,77]
*Akkermansia muciniphila*	Propionate, mucin degradation products	Improves mucus barrier and vagal signaling; inversely correlated with inflammation	Human & animal [78]
*Lactobacillus rhamnosus*, *L. brevis*, *L. helveticus*	GABA, lactate	Modulate vagal tone, stress response, and GABAergic neurotransmission	Human (pilot) & animal [79]
*Bifidobacterium longum*, *B. dentium*	GABA, acetate	Regulate anxiety-like behavior and influence BDNF expression	Human (small trials) & animal [80,81]
*Enterococcus faecalis*, *Streptococcus thermophilus*	Serotonin (5-HT)	Promote tryptophan hydroxylase 1 (TPH1) in enterochromaffin cells → increased gut 5-HT	Animal & in vitro [82,83,84]
*Escherichia coli*, *Bacillus subtilis*	Dopamine, norepinephrine	Produce or induce host catecholamine release; affect gut motility and stress axis	In vitro & animal [82,85]
*Clostridium butyricum*	Butyrate, 5-HT modulation	Reduces microglial activation, improves cognitive performance	Human (small trials) & animal [86,87]
*Candida albicans*	Indirect stimulation of 5-HT release	Alters tryptophan metabolism and gut sensory signaling	In vitro & human correlations [88,89]

**Table 2:** Alterations in gut microbiota composition across NDs: LPS, lipopolysaccharide; SCFA, short-chain fatty acid; Th17, T helper 17 cells; IL, interleukin; AD, Alzheimer disease; ALS, Amyotrophic lateral sclerosis; HD, Huntington disease; MS, Multiple sclerosis; PD, Parkinson disease.

Disease	Microbial Genus/Species		Key Inflammatory/Immune Effects
**PD**	↑ Increased	*Enterobacteriaceae*, *Escherichia/Shigella*, *Bacteroides*, *Ruminococcus*, *Atopobium*, *Desulfovibrio*, *Proteobacteria*, *Lachnospiraceae*, *Alistipes*, *Erysipelotrichaceae*, *Helicobacter pylori*, *Porphyromonas gingivalis*	↑ LPS, bacterial amyloids, and toxins (e.g., fragilysin) → epithelial junction damage → increased gut permeability → systemic inflammation → microglial activation → Aβ aggregation and tau phosphorylation
	↓ Decreased	*Eubacterium rectale*, *Bifidobacterium*, *Dialister*, *Lactobacillus*, *Prevotella*, *Faecalibacterium prausnitzii*, *Bacillota*, *Actinobacteria*	↓ SCFA (butyrate, isobutyrate) and indole derivatives → loss of anti-inflammatory tone → impaired intestinal barrier → enhanced neuroinflammation and oxidative stress
**AD**	↑ Increased	*Escherichia/Shigella*, *Bacteroides*, *Ruminococcus*, *Desulfovibrio*, *Enterobacteriaceae*, *Alistipes*, *Proteobacteria*, *Helicobacter pylori*, *Porphyromonas gingivalis*	↑ LPS and bacterial amyloids → intestinal barrier disruption → systemic inflammation → microglial activation → Aβ and tau aggregation → neuronal loss
	↓ Decreased	*Bifidobacterium*, *Lactobacillus*, *Eubacterium rectale*, *Faecalibacterium prausnitzii*, *Prevotella*, *Bacillota*, *Actinobacteria*	↓ SCFA (butyrate, acetate) → impaired anti-inflammatory signaling and TJ integrity → increased permeability → oxidative stress and cognitive decline
**MS**	↑ Increased	*Akkermansia muciniphila*, *Methanobrevibacter smithii*, *Bacillota (some species)*, *Euryarchaeota*, *Actinobacteria (in obese MS patients)*	↑ Th17 activation and proinflammatory cytokines (IL-17, IL-21, IL-22) → reduced Treg cells → immune imbalance, demyelination, increased intestinal permeability
	↓ Decreased	*Faecalibacterium prausnitzii*, *Bacteroides fragilis*, *Eubacterium rectale*, *Megamonas funiformis*, *Clostridium* cluster XIVa, *Prevotella*, *Bacteroidota*	↓ SCFA producers (butyrate, propionate) → impaired Treg induction → increased neuroinflammation and oxidative stress
**HD**	↑ Increased	*Clostridium (cluster XIVa)*, *Erysipelotrichales*, *Bacteroidales*, *Burkholderiales*, *Lactobacillales*, *Coriobacteriales*	↑ proinflammatory cytokines (IL-6, TNF-α, IL-1β) → intestinal barrier disruption → systemic inflammation → neuroinflammatory signaling and mitochondrial dysfunction
	↓ Decreased	*Eubacterium rectale*, *Bacteroides fragilis*, *Clostridiales (overall)*, *Faecalibacterium*, *Butyrate-producing bacteria*	*↓ SCFA and tryptophan/kynurenine pathway regulation → impaired mitochondrial energy balance*, *excitotoxicity*, *and neuronal degeneration*
**ALS**	↑ Increased	*Ruminococcus*, *Desulfovibrio*, *Bacteroides*, *Clostridium* spp.	↑ LPS and proinflammatory metabolites → intestinal barrier disruption → systemic inflammation → microglial activation and motor neuron degeneration
	↓ Decreased	*Akkermansia muciniphila*, *Butyrivibrio fibrisolvens*, *Anaerostipes*, *Oscillibacter*, *Lachnospiraceae*, *Bacillota* (overall), *Butyrate-producing bacteria*	↓ butyrate and nicotinamide production → metabolic stress → impaired mitochondrial and neuronal energy balance → neuroinflammation and motor neuron loss

## Data Availability

No new data were generated or analyzed in this study. Data sharing is not applicable to this article.

## Abbreviations

5-HT	serotonin
5-HTP	5-hydroxytryptophan
6-OHDA	6-hydroxydopamine
α-SYN	α-synuclein
aMCI	Mild cognitive impairment
Aβ	Amyloid-β
AD	Alzheimer’s disease
ALS	Amyotrophic lateral sclerosis
AMPs	Antimicrobial peptides
ANS	Autonomic nervous system
AhR	Aryl hydrocarbon receptor
APP	amyloid precursor protein
BBB	Blood–brain barrier
BDNF	Brain-derived neurotrophic factor
BMD	Bone mineral density
CNS	Central nervous system
CH25H	Cholesterol 25-hydroxylase
DCs	Dendritic cells
DMV	Dorsal motor nucleus of the vagus
DRG	Dorsal root ganglia
ENS	Enteric nervous system
EAE	Experimental autoimmune encephalomyelitis
FITC	Fluorescein isothiocyanate
FFAR2	free fatty acid receptor 2
FFAR3	free fatty acid receptor 3
FMT	Fecal microbiota transplantation
GALT	Gut-associated lymphoid tissue
GABA	γ-aminobutyric acid
GBA	Gut–brain-axis
GERD	Gastroesophageal reflux
GF	Germ free
GI	Gastrointestinal
GIT	Gastrointestinal tract
Gi/o	inhibitory G protein alpha i/o family
Gq/11	G protein alpha q/11 family
GPCR	G protein-coupled receptor
GPR43	G protein-coupled receptor 43
GPR41	G protein-coupled receptor 41
GPR109A	G protein-coupled receptor 109A
GVB	Gut vascular barrier
GWAS	Genome-wide association studies
HCAR2	hydroxycarboxylic acid receptor 2
HD	Huntington disease
HDAC	Histone deacetylase
HMOs	Human milk oligosaccharides
HTT	Huntington gene
HPA	Hypothalamic–pituitary–adrenal axis
HR	Hazard ratio
LBs	Lewy bodies
LPS	Lipopolysaccharides
MAMPs	Microbe-associated molecular patterns
MGBA	Microbiota-gut–brain axis
MS	Multiple sclerosis
MUC	Transmembrane mucin
NICU	Neonatal intensive care unit
NDs	Neurodegenerative diseases
NLRP3	NOD-like receptor family pyrin domain-containing 3
NTS	Nucleus tractus solitarius
PD	Parkinson’s disease
PFFs	Preformed fibrils
PGN	Peptidoglycan
PRRs	Pattern recognition receptors
PSEN1	Presenilin 1
PYY	peptide YY
QS	Quorum sensing
RgpA	Releases gingipain
ROS	Reactive oxygen species
RRMS	Relapsing-remitting MS
RBD	REM sleep behavior disorder
SPMS	Secondary progressive MS
SCFAs	Short-chain fatty acids
SIBO	Small intestinal bacterial overgrowth
SNpc	Substantia nigra pars compacta
SG	Sympathetic ganglia
TCA	Tricarboxylic acid cycle
TFHs	T follicular helper cells
TJ	Tight junction
TLRs	Toll-like receptors
TMAO	Trimethylamine N-oxide
TPH1	Tryptophan hydroxylase 1
Treg	Impaired regulatory T cell
IFN-I	Type I interferon
VGLUT1	Vesicular glutamate transporter 1
VN	Vagus nerve
VNS	Vagus-nerve stimulation
ZO	zonula occluden
